# A Robust Numerical Framework for Hollow-Fiber Membrane Module Simulation and Solver Performance Analysis

**DOI:** 10.3390/membranes16040154

**Published:** 2026-04-21

**Authors:** Diego Queiroz Faria de Menezes, Marília Caroline Cavalcante de Sá, Nayher Andres Clavijo Vallejo, Thainá Menezes de Melo, Luiz Felipe de Oliveira Campos, Thiago Koichi Anzai, José Carlos Costa da Silva Pinto

**Affiliations:** 1 Programa de Engenharia Química, Instituto Alberto Luiz Coimbra de Pós-Graduação e Pesquisa de Engenharia (COPPE), Universidade Federal do Rio de Janeiro, Rio de Janeiro 21941-972, RJ, Brazilpinto@peq.coppe.ufrj.br (J.C.C.d.S.P.); 2 Centro de Pesquisas Leopoldo Américo Miguez de Mello (CENPES), Petrobras, Rio de Janeiro 21941-915, RJ, Brazil

**Keywords:** hollow fiber membranes, membrane modeling, orthogonal collocation, pseudotransient method, natural gas separation

## Abstract

Robust numerical frameworks are essential for the simulation, design, monitoring, and control of membrane-based separation units, particularly under highly nonlinear and industrially relevant operating conditions. In this context, a comprehensive phenomenological and numerical framework is proposed for the simulation of hollow-fiber membrane modules, incorporating coupled mass, momentum (through pressure drop), and energy transport equations. The governing equations are discretized using a rigorous orthogonal collocation formulation, and the performances of two numerical solution strategies are systematically investigated for the first time to allow the in-line and real-time implementation of the model: a steady-state approach based on the Newton–Raphson method with careful treatment of initial estimates, and a pseudotransient formulation. Particularly, an original and consistent numerical treatment is introduced for the energy balance at boundaries where the permeate flow vanishes, enabling the stable incorporation of thermal effects and Joule–Thomson phenomena. The results clearly show that the steady-state Newton–Raphson approach provides the best overall performance in terms of computational efficiency, numerical robustness, and accuracy when physically consistent initial profiles are employed. In particular, the combination of a linear initial guess and a numerical mesh constituted of four collocation points yielded the most favorable balance between convergence speed, numerical robustness, and accuracy for the base-case sensitivity analysis. For monitoring-oriented applications, the numerical choice should be weighted primarily toward computational performance once physical consistency and convergence criteria are satisfied, rather than toward maximum mesh-refinement accuracy. In this context, small differences in internal-fiber profiles can be compensated through real-time permeance estimation and are negligible when compared with measurement uncertainty in real industrial processes. Under extreme operating conditions involving low concentrations, low flow rates, and highly permeable species, the pseudotransient formulation proved to be a reliable auxiliary strategy, enabling robust convergence when suitable initial guesses were not readily available. The proposed framework is validated against experimental data from the literature and subjected to extensive convergence and sensitivity analyses, providing a reliable basis for simulation and for assessing computational feasibility in in-line and real-time monitoring-oriented applications. A full demonstration of digital-twin integration, online parameter updating, reduced-order coupling, and closed-loop control is beyond the scope of the present study and will be addressed in future work.

## 1. Introduction

Natural gas represents a significant fraction of the currently used fuels. Despite being derived from fossil sources, natural gas is normally regarded as a relatively clean fuel, as methane combustion emits 30% less carbon dioxide during combustion, when compared to liquid oil fuels [1]. Moreover, natural gas can also be obtained from renewable sources [2,3]. Nevertheless, besides methane, fossil-based natural gas contains various contaminants, with ${\mathrm{CO}}_{2}$ and $H_{2}S$ usually being the most concentrated ones. ${\mathrm{CO}}_{2}$, when released into the atmosphere, significantly contributes to the greenhouse effect and, if it remains in the natural gas stream, can cause corrosion in equipment and pipelines in the presence of moisture [4]. $H_{2}S$, on the other hand, is a notorious toxic gas [5].

Based on the previous comments, natural gas purification must be carried out to remove contaminants and meet gas combustion specifications. Historically, chemical absorption with alkaline substances has been the most widely used natural gas purification method. However, the costs associated with this technique increase proportionally to the amount of contaminants present in the gas [1], demanding the use of larger process vessels and higher amounts of chemicals. For these reasons, separation units based on membranes constitute excellent alternatives to the traditional method, both from the environmental and operational perspectives. In particular, the use of membranes can allow for more efficient contact between the gas and the absorbing liquid, reducing the amount of solvent required and, consequently, the costs associated with chemical absorption. However, the use of membrane separation units based on gas absorption does not completely eliminate the use of solvents and absorbing compounds and their inherent consequences.

Since the 1970s [6], the use of polymeric membranes for the direct separation of gaseous streams has become a viable alternative. According to this mechanism, acid gases are separated from natural gas due to differences in both membrane affinity (solubility) and molecular mobility within the polymer matrix (diffusivity), while the transmembrane pressure difference provides the driving force for permeation. In fact, different types of polymeric membranes are available in the market to remove acid gases from gaseous streams, based on distinct materials and presenting different geometric features for gas exchange. For example, spiral-type membranes were the first to be developed [6] and are still in use today. However, hollow fiber membranes allow for a larger exchange area with a higher degree of packing, enabling the use of smaller separation equipment [7]. Additionally, these membranes are less susceptible to flooding due to condensation inside the module, which can occur at temperatures below the dew point.

The use of mathematical models to represent gas separation processes constitutes a powerful tool for simulating different operating conditions, as well as for designing new units, evaluating operational costs, and monitoring processes. Additionally, these models can be of fundamental importance for the development of digital twins and allow the advanced monitoring and control of real industrial units. In this context, to be of practical use, the modeling approach must describe the transport equations and explicitly provide the permeate flow through the membrane and pressure drop throughout the module [8].

Ref. [8] developed a hollow-fiber membrane model that describes the detailed permeate and retentate compositions, as well as the pressure drop along the fiber. The resulting formulation is a coupled nonlinear system with 2(N−1)+4 equations for the isothermal case and 2N+4 equations for the non-isothermal case, where *N* is the number of components. This system can be solved by different methods. Pan [9] presented an experimentally validated multicomponent gas permeation model with hydrogen recovery and acid-gas separation, which has been widely used for validation of more recent models [7,10]. The authors used the shooting method to solve the obtained system of equations, as the boundary conditions are defined at distinct points of the separation unit, thus requiring an iterative solution of the model equations. However, this method may present convergence problems [8], especially in non-isothermal multicomponent systems with pressure- and temperature-dependent permeabilities, which is particularly problematic for online and real-time applications.

One of the main uses of the orthogonal collocation technique is to reduce the system of ordinary differential equations to a lower-dimensional system of ordinary differential equations or to a system of nonlinear algebraic equations [11]. Furthermore, it has also been widely used to solve separation units based on hollow fiber membranes [12]. This dimensionality reduction characteristic is particularly useful when the process model is embedded in optimization layers, as reported in studies of distillation column optimization and in nonlinear model predictive control (NMPC) formulations, where a lower computational cost can be achieved without significant loss of predictive accuracy [11,13]. Chu et al. [14] lists several authors who have used this technique, highlighting the advantage of obtaining profiles of variables of interest along the fiber length. To solve the system of ordinary differential equations obtained after application of orthogonal collocation, the Newton–Raphson (NR) method is commonly employed to ensure the convergence of the flow profiles. This method is extremely popular, with numerous theoretical and applied developments over the years [15]. As it is well known, the NR method employs a first-order approximation in the vicinity of the approximate solution, generating a linear system of equations that must be solved iteratively to reach the final solution [16].

Although the NR method is frequently very efficient, there are cases where it performs poorly. For instance, when there is an inflection point close to the root, the NR method can lead to divergence and oscillatory responses near local maxima and minima. Moreover, if local derivatives are small and close to zero, the NR method can lead to solution estimates that are located far from the region of interest, demanding the inversion of nearly singular matrices and provoking the occurrence of serious numerical and convergence problems [17].

The pseudotransient method is an alternative numerical scheme that can be used to ensure the convergence of the differential equation system [18]. According to this method, the balance equations are described in dynamic form, and the simulation is carried out until temporal convergence is achieved. In this case, time can be regarded as an iteration parameter and the dynamic equations can be written in a simplified form only to guarantee that the trajectory will lead to the desired steady-state solution [19]. Several authors have used this method to overcome issues that arise when using the Newton–Raphson method, such as initial guess problems [20] and decoupling of the equation system, while simultaneously mitigating the development of iterative oscillatory solutions [21].

While significant progress has been made in the modeling of hollow-fiber membrane systems, important limitations remain when thermal effects and numerical robustness are considered simultaneously. Early studies such as Kaldis et al. [22] established efficient orthogonal collocation frameworks based on Jacobi polynomials, but were restricted to simplified, isothermal, and binary systems. Later, Chu et al. [14] extended these formulations to multicomponent systems, although still neglecting thermal coupling and without fully detailing the numerical construction of the collocation framework.

More recently, Helmersen [23] incorporated energy balances into the model, highlighting the relevance of thermal effects but also reporting numerical instabilities and convergence difficulties associated with the coupled system. These observations indicate that the inclusion of energy transport introduces additional numerical challenges that are not fully addressed in the existing literature.

In addition to these methodological limitations, most available models are not designed for real-time applications, in which computational efficiency, numerical robustness, and predictive accuracy must be achieved simultaneously. As a result, the direct use of these models in monitoring, parameter estimation, and soft-sensor applications remains limited.

As a practical benchmark for real-time monitoring at a 5-min sampling frequency (300 s), recent industrial applications of detailed membrane models report full-unit simulation times of approximately 13 s [24]. In that work, real-time monitoring of ${\mathrm{CO}}_{2}$ removal from natural gas is performed using spiral-wound dense membranes, with the entire industrial unit being modeled, simulated, and optimized, including multiple modules in series and parallel tube arrangements. Notably, even at the full-unit scale, the optimization procedure was shown to converge in most cases within the 5-min sampling interval, highlighting the feasibility of integrating detailed membrane models into real-time monitoring and optimization frameworks.

In this context, there is a clear need for a modeling and numerical framework that simultaneously addresses thermal coupling, numerical stability, and computational performance.

Based on the gaps identified in the previous discussion, the present work proposes a comprehensive phenomenological model to represent gas separation in hollow-fiber membrane modules. In particular, the present work extends the formulation of the energy balance by accounting for thermal exchanges associated with mass transfer through the membrane and the Joule–Thomson effect in hollow fiber modules. In order to solve the model equations, the ordinary differential equations are discretized using the orthogonal collocation method originally proposed by Chu et al. [14], and an original and consistent numerical treatment is proposed to address the boundary condition of the energy balance at locations where the permeate flow vanishes.

Additionally, the performance of numerical strategies based on steady-state Newton–Raphson procedures and pseudotransient techniques is systematically investigated and compared within a unified framework, with explicit focus on real-time and in-line applicability.

In this context, the proposed framework is assessed in terms of computational efficiency, robustness, and accuracy, aiming to meet the requirements of real-time optimization and monitoring tasks. The model is further employed to explore different operating regimes and gas composition ranges, enabling the identification of conditions under which the convergence of steady-state solvers becomes sensitive to initialization, potentially impacting real-time model responses.

Section 2 begins by revisiting the model proposed by Chu et al. [14], highlighting key aspects related to the formulation of the mass-balance equations. Subsequently, the development of the energy balance is presented in an original form for this class of applications. The numerical framework based on orthogonal collocation is then described and combined with the Newton–Raphson and pseudotransient approaches to obtain steady-state solutions of the governing equations. The section concludes with the presentation of the proposed numerical treatment for the energy-balance boundary condition.

In the Section 3, the numerical performance of the Newton–Raphson and pseudotransient approaches are compared through convergence, sensitivity, and mesh refinement analyses. To the best of the authors’ knowledge, this work provides the first systematic and quantitative comparison between these solution strategies within a unified orthogonal collocation framework for hollow-fiber membrane module simulation. The model is validated against literature data for both mass and energy balances, and the computational costs associated with the steady-state and pseudotransient solution strategies are assessed. Finally, the main conclusions are drawn based on the obtained results.

## 2. Methodology

### 2.1. Model

#### 2.1.1. Mass Balance Equations

The mathematical model developed in the present work is based on the modeling and simulation strategies presented by Chu et al. [14] and Helmersen [23]. Chu et al. [14] developed a model for the mass balance in hollow-fiber membranes based on Fick’s Law (sorption-diffusion phenomena). The differential equations generated by the modeling were discretized by orthogonal collocation, resulting in a system of nonlinear algebraic equations, which was solved with the Newton–Raphson method. The authors called this model the Mollocator and compared the results with those provided by the ChemBrane model, based on the work by Grainger [25], under two experimental scenarios. It is important to highlight that one relevant difference between the model proposed by Chu et al. [14] and that proposed by Helmersen [23] is the treatment of the pressure drop on the shell side. In the present work, pressure drop effects are considered only on the permeate side (fiber lumen), while shell-side pressure variations are neglected based on typical industrial operating conditions.

In summary, the model proposed here adopts the following assumptions:

1. Permeation rates and mass transfer obey Fick’s law and the solution-diffusion mechanism.
2. Plug flow is assumed along the axial direction, without radial components. This assumption is justified by the high length-to-diameter ratio of hollow fibers, which promotes predominantly axial flow and minimizes dispersion. Additionally, the typically laminar flow regime suppresses radial mixing, while the confined geometry prevents significant radial convection. As a result, mass transfer in the radial direction occurs mainly by diffusion and permeation through the membrane, allowing the system to be accurately described as one-dimensional.
3. Heat and mass transfer resistances are concentrated in the membrane, so that boundary layer resistances are assumed to be negligible. This assumption is justified by the small characteristic dimensions and high specific area of hollow fibers, which lead to thin boundary layers and enhanced convective transport on both sides of the membrane. As a result, external heat and mass transfer resistances are significantly lower than the intrinsic membrane resistance, allowing the overall transport to be effectively controlled by the membrane itself.
4. The permeances of the components are constant. This assumption is justified for the present study, as it considers a narrow operating range of pressure and temperature, under which gas solubility and diffusivity in the membrane remain nearly unchanged. However, in real applications operating at higher pressures, over wider temperature ranges, or under conditions prone to plasticization and competitive sorption, permeance may vary significantly and should be modeled as a function of pressure and temperature.
5. Sweep gas is not considered in the permeate stream.
6. The effective thickness of the membrane is uniform throughout the fiber.
7. Pressure drop in the axial direction is considered only within the fiber (permeate side). Pressure variations along the shell side are assumed to be negligible, based on typical industrial operating conditions where the feed flow is distributed over a large cross-sectional area, resulting in relatively small pressure gradients compared to those inside the fiber lumen.
8. Polarization effects are not considered on either side. This assumption is justified by the small characteristic dimensions and high specific area of hollow fiber modules, which lead to thin boundary layers and high mass transfer coefficients, minimizing concentration gradients near the membrane surface.

**Figure 1:**
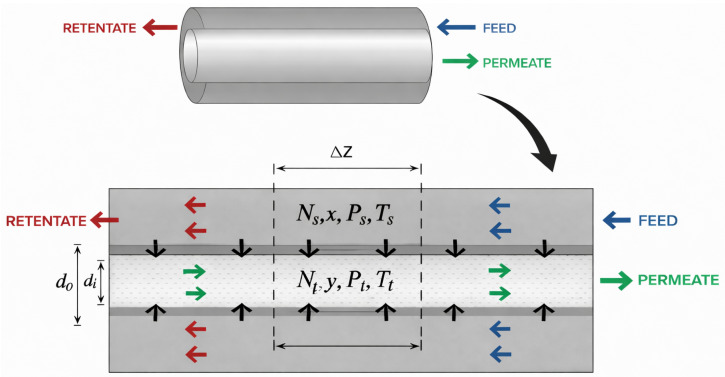
Schematic representation of gas permeation in a hollow-fiber membrane under countercurrent flow, with the feed on the shell side. The permeate side at z=0 is a closed-end boundary (dead end), with no purge-gas flow. Blue arrows indicate the feed stream, green arrows indicate the permeate stream, red arrows indicate the retentate stream, and the figure background is white.

Figure 1 presents the schematic representation of the membrane operation in countercurrent mode, in which the high-pressure multicomponent gas is fed into the shell side and flows toward the negative *z* direction. The components permeate through the membrane, where the permeated gas flows countercurrent to the shell-side flow toward the positive *z* direction.

Further details regarding the development of mass balances and momentumbalances are presented elsewhere [23]. Only the pressure drop along the permeate side is explicitly modeled, while the shell-side pressure is assumed constant along the axial direction. Briefly, considering the control volume on the infinitesimal element Δz and assuming a steady state, the following equations were obtained for the molar flow rates on the feed and permeate sides and for the pressure drop on the permeate side:

(1) $$\frac{dN_{s}x_{i}}{dz}=n_{fb}\;\cdot \;\pi \;\cdot \;d_{o}\;\cdot \;J_{i}$$

(2) $$\frac{dN_{t}y_{i}}{dz}=n_{fb}\;\cdot \;\pi \;\cdot \;d_{o}\;\cdot \;J_{i}$$

(3) $$\frac{dP_{t}}{dz}=\frac{128\;\cdot \;\eta \;\cdot \;R\;\cdot \;T_{t}}{n_{fb}\;\cdot \;\pi \;\cdot \;d_{i}^{4}\;\cdot \;P_{t}}\sum_{i=1}^{nc}N_{t}y_{i}$$

where $N_{s}$, $N_{t}$, $P_{t}$, and $J_{i}$ are the molar flow rate on the feed side (shell side) [$\frac{\mathrm{mol}}{s}$], the molar flow rate inside the fiber [$\frac{\mathrm{mol}}{s}$], the pressure within the fiber [Pa] and the molar flux through the membrane [$\frac{\mathrm{mol}}{s\;\cdot \;m^{2}}$], respectively. η, $T_{t}$, $n_{fb}$, and nc are the permeate viscosity [Pa·s], the temperature inside the fiber [K], the number of fibers in the module [-], and the number of components that permeate through the membrane [-], respectively. $d_{o}$ and $d_{i}$ are the external and internal diameters of the fiber [m], respectively. The molar flux through the membrane is defined as follows:

(4) $$J_{i}=P_{i}\;\cdot \;\left(P_{s}\;\cdot \;x_{i}-P_{t}\;\cdot \;y_{i}\right),\;P_{i}=\frac{\Pi_{i}}{\delta },\;\Pi_{i}=D_{i}\;\cdot \;K_{i}$$

where $D_{i}$ is the diffusivity of component *i*, $K_{i}$ is the sorption coefficient of component *i*, δ is the thickness of the membrane, $\Pi_{i}$ is the permeability of component *i*, and $P_{i}$ is the permeance of component *i*. The mole fractions on the feed side, $x_{i}$, and on the permeate side, $y_{i}$, are defined as follows:

(5) $$x_{i}=\frac{N_{s,i}}{N_{s}},\;y_{i}=\frac{N_{t,i}}{N_{t}}$$

where $N_{s,i}$ and $N_{t,i}$ correspond to the molar flow rates of component *i* on the shell and tube sides, respectively.

In order to generate the dimensionless model variables for purposes of normalization, ease of generalization, and numerical implementation, the following variable transformations are proposed:

(6) $$z^{*}=\frac{z}{L},\;{\left(N_{s}x_{i}\right)}^{*}=\frac{N_{s}x_{i}}{\sum_{i=1}^{nc}{\left(N_{s}x_{i}\right)}_{f}},\;{\left(N_{t}y_{i}\right)}^{*}=\frac{N_{t}y_{i}}{\sum_{i=1}^{nc}{\left(N_{s}x_{i}\right)}_{f}},\;P_{s}^{*}=\frac{P_{s}}{P_{sf}},\;P_{t}^{*}=\frac{P_{t}}{P_{sf}}$$

Thus, the set of equations in dimensionless form is given by Equations (7)–(9).

(7) $$\frac{d{\left(N_{s}x_{i}\right)}^{*}}{dz^{*}}=K_{m}P_{i}\left[P_{s}^{*}\frac{{\left(N_{s}x_{i}\right)}^{*}}{\sum_{i=1}^{nc}{\left(N_{s}x_{i}\right)}^{*}}-P_{t}^{*}\frac{{\left(N_{t}y_{i}\right)}^{*}}{\sum_{i=1}^{nc}{\left(N_{t}y_{i}\right)}^{*}}\right]$$

(8) $$\frac{d{\left(N_{t}y_{i}\right)}^{*}}{dz^{*}}=K_{m}P_{i}\left[P_{s}^{*}\frac{{\left(N_{s}x_{i}\right)}^{*}}{\sum_{i=1}^{nc}{\left(N_{s}x_{i}\right)}^{*}}-P_{t}^{*}\frac{{\left(N_{t}y_{i}\right)}^{*}}{\sum_{i=1}^{nc}{\left(N_{t}y_{i}\right)}^{*}}\right]$$

(9) $$\frac{dP_{t}^{*}}{dz^{*}}=K_{pt}\frac{\sum_{i=1}^{nc}{\left(N_{t}y_{i}\right)}^{*}}{P_{t}^{*}}$$

where the coefficients $K_{m}$ and $K_{pt}$ are defined as follows:

(10) $$K_{m}=\frac{\pi \;\cdot \;L\;\cdot \;n_{fb}\;\cdot \;d_{o}\;\cdot \;P_{sf}}{\sum_{i=1}^{nc}{\left(N_{s}x_{i}\right)}_{f}}$$

(11) $$K_{pt}=\frac{128\;\cdot \;L\;\cdot \;\eta \;\cdot \;R\;\cdot \;T_{tf}\;\cdot \;\sum_{i=1}^{nc}{\left(N_{s}x_{i}\right)}_{f}}{P_{s,f}^{2}\;\cdot \;n_{fb}\;\cdot \;\pi \;\cdot \;d_{i}^{4}}$$

The boundary conditions of the dimensionless system are given by Equation (12).

(12) $$\left\{\begin{array}{cc} z^{*}=0 & z^{*}=1\\ {\left(N_{t}y_{i}\right)}^{*}=0 & {\left(N_{s}x_{i}\right)}^{*}={\left(N_{s}x_{i}\right)}_{f}^{*}\\ \; & P_{t}^{*}=P_{tp}^{*} \end{array}\right.$$

where $P_{tp}^{*}=P_{tp}/P_{sf}$ is the dimensionless permeate outlet pressure.

On the other hand, when the membrane operates in co-current flow, the corresponding boundary conditions are given by Equation (13):

(13) $$\left\{\begin{array}{cc} z^{*}=0 & z^{*}=1\\ {\left(N_{t}y_{i}\right)}^{*}=0 & \\ {\left(N_{s}x_{i}\right)}^{*}={\left(N_{s}x_{i}\right)}_{f}^{*} & \\ \; & P_{t}^{*}=P_{tp}^{*} \end{array}\right.$$

It is worth mentioning that, in the following sections, the equations are presented using the nomenclature and indexing adopted for the countercurrent configuration. The corresponding expressions for co-current operation can be found in Chu et al. [14].

#### 2.1.2. Energy Balance Equations

The proposed gas separation model also comprises the overall energy balance. Additionally, it includes constitutive equations that account for the solution-diffusion permeation mechanism and equations of state to describe the Joule–Thomson effect. Several assumptions were made for the development of the energy-balance model, taking into account the thermal effects present in the gas permeation process:

1. The heat loss to the surroundings is negligible;
2. The radial effects are neglected [26,27];
3. Feed composition and temperature are uniform;
4. The thermodynamic behavior of the gas can be described by virial correlations [27];
5. The permeate stream is subject to temperature changes due to the Joule–Thomson effect.

According to the principle of energy conservation, heat transport occurs due to both mass transfer and the temperature difference between the retentate and permeate streams, and considering that both streams flow in the z-direction, the following can be written:

(14) $$\sum_{i}{\left(N_{s}x_{i}\right)}^{*}\;\cdot \;c_{p_{i}}^{*}\;\cdot \;\frac{dT_{s}^{*}}{dz^{*}}=\lambda \;\cdot \;\left(T_{s}^{*}-T_{t}^{*}\right)$$

(15) $$\sum_{i}{\left(N_{t}y_{i}\right)}^{*}\;\cdot \;c_{p_{i}}^{*}\;\cdot \;\frac{dT_{t}^{*}}{dz^{*}}=\gamma \;\cdot \;\sum_{i}c_{pi}^{*}\;\cdot \;\mu_{i}^{*}\;\cdot \;P_{i}^{*}\;\cdot \;\left(P_{s}^{*}x_{i}-P_{t}^{*}y_{i}\right)+\lambda \;\cdot \;\left(T_{s}^{*}-T_{t}^{*}\right)$$

where ${\left(N_{s}x_{i}\right)}^{*}$, ${\left(N_{t}y_{i}\right)}^{*}$, $P_{s}^{*}$, $P_{t}^{*}$, and $z^{*}$ are previously defined in Equation (6). The additional dimensionless variables and parametric groups required by the energy balance are given below:

(16) $$T_{s}^{*}=\frac{T_{s}}{T_{f}},\;T_{t}^{*}=\frac{T_{t}}{T_{f}},\;c_{pi}^{*}=\frac{c_{p_{i}}}{c_{p_{m}}},\;\mu_{i}^{*}=\frac{\mu_{i}}{\mu_{f}},\;P_{i}^{*}=\frac{P_{i}}{P_{m}}$$

(17) $$\lambda =\frac{n_{fb}\;\cdot \;\pi \;\cdot \;d_{o}\;\cdot \;L\;\cdot \;U}{\sum_{i}{\left(N_{s}x_{i}\right)}_{f}\;\cdot \;c_{pm}},\;\gamma =\frac{n_{fb}\;\cdot \;\pi \;\cdot \;d_{o}\;\cdot \;\Delta P\;\cdot \;L\;\cdot \;P_{f}\;\cdot \;\mu_{f}\;\cdot \;P_{m}}{\sum_{i}{\left(N_{s}x_{i}\right)}_{f}\;\cdot \;T_{f}}$$

In Equations (16) and (17), the variables that were not previously defined are *U*, the global heat transfer coefficient across the membrane barrier [$\frac{J}{s\;\cdot \;m^{2}\;\cdot \;K}$]; P, the permeance [$\frac{\mathrm{mol}}{m^{2}\;\cdot \;s\;\cdot \;\mathrm{Pa}}$]; $c_{p}$, the heat capacity [$\frac{J}{\mathrm{mol}\;\cdot \;K}$]; and μ, the Joule–Thomson coefficient [$\frac{K}{\mathrm{Pa}}$]. In addition, ΔP denotes the pressure difference between the streams, and the subscript *m* denotes the base component.

The balance equations described above correspond to a set of first-order ordinary differential equations with the following boundary conditions defined in Equation (18).

(18) $$\left\{\begin{array}{cc} z^{*}=0 & z^{*}=1\\ T_{t}^{*}=T_{t0}/T_{f} & T_{s}^{*}=T_{f}/T_{f} \end{array}\right.$$

where $T_{t0}$ is the temperature of the permeate stream at the membrane point where there is no permeate flow. Therefore, this is a value calculated by an iterative method, starting from an initial value provided by the Joule–Thomson effect. More details on the methodology for calculating $T_{t0}$ are presented in the following section. Particularly, this constitutes an original numerical approach that is needed to take the Joule–Thomson effect into consideration.

#### 2.1.3. Boundary Condition Treatment

As shown in Section 2.1.2, the energy balance of the system is described by a set of first-order ordinary differential equations with the boundary conditions defined in Equation (18). As one can see, at the specification of the boundary condition at $z^{*}=0$, the dimensionless temperature $T_{t}^{*}$ is expressed as a function of $T_{t0}$ (the temperature of the permeate stream at the membrane point with null permeate flow). However, the specification of this temperature constitutes a drawback since this kind of information is not typically available from the operational conditions of the separation system, implying impractical measures or additional numerical efforts.

Since the system of mass-balance equations and the system of energy-balance equations can be solved sequentially, an intermediate iterative numerical step is proposed to estimate $T_{t}^{*}$ based on the proper temperature profile of the membrane. The iterative procedure considers the occurrence of a smooth temperature profile, so that oscillating behavior or abrupt changes along the axial direction should not take place.

It was observed that inaccurate specifications of $T_{t0}$ or $T_{t}^{*}$ at z=0 can lead to oscillatory behaviors of the temperature profiles of the membrane due to instabilities of the solution method. Therefore, it is assumed that the correct value of the boundary condition $T_{t}^{*}$ at z=0 must necessarily generate smooth profiles when the entire system is solved. The iterative process considers as an initial guess the value provided by the Joule–Thomson effect in the energy-balance equation, presented by Equation (19).

(19) $$T_{t0}=T_{f}+\mu \left(P_{t}-P_{s}\right)$$

The iterative procedure can also be defined as an optimization problem:

(20) $$\begin{array}{cc} \min_{T_{t0}}\; & \sum_{i=1}^{N_{peaks}}h_{peaks}\\ s.t.\; & T_{t0_{min}}\leq T_{t0}\leq T_{t0_{max}}\\ \; & T_{t}^{*}>0 \end{array}$$

where $h_{peaks}$ corresponds to oscillation amplitudes after excluding noisy fluctuations of numerical origin. In this work, peak amplitude is computed from maxima and minima in the time series using two approaches:

- Simple approach: minima are identified as Y[t−1]>Y[t]<Y[t+1] and maxima as Y[t−1]<Y[t]>Y[t+1]. A peak is considered valid when the difference between a minimum and the subsequent maximum is greater than a tolerance $h_{tol}$ [28].
- Prominence approach: peaks are identified and filtered by minimum prominence. In this context, prominence measures how much a peak stands out relative to its surrounding baseline (defined from the left and right bases). The method returns peak indices and their prominences; only peaks with prominence above a prescribed threshold are retained, and $h_{peaks}$ is computed from these filtered oscillations.

Thus, the solution procedure of the complete model for describing hollow-fiber membranes consists first in solving the mass balance, followed by the solution of the energy balance, which internally performs an iterative step to determine the boundary condition at z=0.

The optimization objective based on the sum of peak amplitudes is adopted as a practical smoothness criterion: physically admissible temperature fields are expected to vary continuously along the fiber, whereas spurious oscillations are interpreted as numerical artifacts induced by inconsistent boundary guesses. In practice, the procedure showed stable convergence from Joule–Thomson-based initial values and bounded searches, and no multiple solutions were observed for the tested operating conditions. Although this does not constitute a formal uniqueness proof, it provides numerical evidence of robustness within the investigated scenario space.

### 2.2. Numerical Approaches

#### 2.2.1. Orthogonal Collocation to the Steady-State Case

The system consisting of Equations (7)–(9) describes the variations in molar flows on the shell side, on the fiber side, and the pressure variation along the spatial variable *z*, respectively. Furthermore, the model-specific boundary conditions in Equation (12) must be observed. However, to transform the boundary value problem into a nonlinear algebraic system, the orthogonal collocation method was selected [29,30]. By applying the orthogonal collocation method, first-order differential terms can be approximated as follows:

(21) $${\left(\frac{dY}{dz^{*}}\right)}_{z_{k}^{*}}=\sum_{j=0}^{nz+1}A_{kj}Y_{j}\;,k=0,1,2,3,\ldots ,nz+1$$

where $A_{kj}$ is the matrix of first-order derivatives of the Lagrange interpolating polynomials, concerning the interpolating polynomial *j* and calculated at the collocation point $z_{k}^{*}$; $Y_{j}$ is the set of dimensionless function variables, calculated at the nodal point *j*; the indices *k* and *j* represent the number of nodal points or interpolating polynomials; and $z_{k}^{*}$ represents the *k*-th root of the Jacobi polynomial, with parameters α=0 and β=0 (i.e., the Legendre polynomial), in the range of the dimensioned *z* variable [0–1]. The residual equations of the interpolation approximations, derived from Equations (7)–(9), result in the following:

(22) $$R_{ki}=\sum_{j=0}^{nz}A_{kj}{\left(N_{s}x_{i}\right)}_{j}^{*}+A_{k,nz+1}\;\cdot \;{\left(N_{s}x_{i}\right)}_{f}^{*}-K_{m}P_{i}\left[P_{s}^{*}\frac{{\left(N_{s}x_{i}\right)}^{*}}{\sum_{i=1}^{nc}{\left(N_{s}x_{i}\right)}^{*}}-P_{t}^{*}\frac{{\left(N_{t}y_{i}\right)}^{*}}{\sum_{i=1}^{nc}{\left(N_{t}y_{i}\right)}^{*}}\right]$$

(23) $$R_{ki}=\sum_{j=1}^{nz+1}A_{kj}{\left(N_{t}y_{i}\right)}_{j}^{*}+A_{k0}\;\cdot \;0-K_{m}P_{i}\left[P_{s}^{*}\frac{{\left(N_{s}x_{i}\right)}^{*}}{\sum_{i=1}^{nc}{\left(N_{s}x_{i}\right)}^{*}}-P_{t}^{*}\frac{{\left(N_{t}y_{i}\right)}^{*}}{\sum_{i=1}^{nc}{\left(N_{t}y_{i}\right)}^{*}}\right]$$

(24) $$R_{k}=\sum_{j=0}^{nz}A_{kj}{\left(P_{t}^{*}\right)}_{j}+A_{k,nz+1}\;\cdot \;P_{tp}^{*}-K_{pt}\frac{\sum_{i=1}^{nc}{\left(N_{t}y_{i}\right)}^{*}}{P_{t}^{*}}$$

Finally, the residue Equations (22)–(24) form a nonlinear algebraic system, which can be solved with the Newton–Raphson method. The number of nonlinear algebraic equations to be solved corresponds to (2·nc+1)·(nz+1), where nc is the number of chemical species that permeate the membrane and nz is the total number of collocation points.

#### 2.2.2. Orthogonal Collocation to the Pseudotransient Case

In general, the methods used to solve systems of nonlinear algebraic equations are based on the Newton–Raphson method, as in most cases these methods are very fast and accurate when the initial guess is close to the solution [19]. On the other hand, these methods are highly dependent on the initial guess provided to achieve adequate convergence to the desired solution. Even so, these methods may sometimes present slow convergence or even diverge and lead to physically infeasible solutions. These last two behaviors tend to occur mainly in large equation systems with a high degree of nonlinearity, as in the present case [31].

There are alternative approaches to overcome the disadvantages of Newton–Raphson-type methods, such as the homotopy continuation method and the pseudotransient method, the latter being more commonly used in computational fluid dynamics [32]. In short, the pseudotransient method solves a non-stationary problem that is equivalent to the steady-state problem, transforming the purely nonlinear algebraic system into a system of differential equations. Therefore, when solving the transient problem until attainment of the steady state, time plays the role of a relaxation parameter in the iteration (explicit relaxation), allowing it to reach the feasible, stabilized, and gradual steady-state solution, regardless of any reasonable starting solution [19,33,34].

Gupta et al. [35] applied the pseudotransient method to simulate steady-state flows in cavitation problems with coupled mass and momentum equations. Two cases were studied to illustrate the robustness of the method: (i) flow through a cavitating orifice, leading to a cavitation cloud; (ii) flow in a fuel injector, demonstrating the robustness of the method and its accuracy for a multiphase system with mass transfer. In fact, the pseudotransient method can improve the numerical robustness of steady-state system simulations by improving the diagonal dominance of the Jacobian matrix (with the increased Jacobian) and providing local under-relaxation through the pseudo-time-step size [19,35]. Räss et al. [36] used the pseudotransient method to solve the ice deformation model, with implicit thermomechanical coupling between the equations of ice motion and temperature. The results obtained with the proposed model based on a pseudotransient solution were compared with results provided by a code used for solving the Stokes equations and based on a finite element scheme, indicating the good agreement between the results and the appropriate performance of the proposed pseudotransient method.

Therefore, the pseudotransient method was adopted as a possible numerical strategy to solve the hollow-fiber membrane model, with coupled mass balance and momentum balance. Thus, the transient terms of the method can be included in Equations (22)–(24) (countercurrent case), by making $z^{*}=Z$, ${\left(N_{s}x_{i}\right)}^{*}=X_{i}\left(Z,t\right)$, ${\left(N_{t}y_{i}\right)}^{*}=Y_{i}\left(Z,t\right)$, $P_{s}^{*}=P$, $P_{t}^{*}=p\left(Z,t\right)$, and $K_{m}\beta_{i}=K_{i}$. Furthermore, it is necessary to define the appropriate Boundary Conditions (${\mathrm{BC}}_{s}\to X_{i}\left(Z_{nz+1},t\right)=X_{i}^{f}$, ${\mathrm{BC}}_{t}\to Y_{i}\left(Z_{0},t\right)=0$, and ${\mathrm{BC}}_{p}\to p\left(Z_{nz+1},t\right)=\frac{P_{t}}{P_{sf}}$) and the Initial Conditions (IC: $X_{i}\left(Z_{k},t=0\right)=1$, $Y_{i}\left(Z_{k},t=0\right)=0$, and $p\left(Z_{k},t=0\right)=0$), eliminating 2·nc+1 equations ($R_{nz+1,i}^{s}$, $R_{0,i}^{t}$, and $R_{nz+1}^{p}$). Thus, the new differential algebraic system can be described by Equations (25)–(27).

(25) $$\begin{array}{cc} \tau_{x}\frac{dX_{i}\left(Z_{k},t\right)}{dt}= & +\sum_{j=0}^{nz}A_{kj}\;\cdot \;X_{i}\left(Z_{j},t\right)+A_{k,nz+1}\;\cdot \;{\mathrm{BC}}_{s}+\\ & -K_{i}\left[P\left(\frac{X_{i}\left(Z_{k},t\right)}{\sum_{i=1}^{nc}X_{i}\left(Z_{k},t\right)}\right)-p\left(Z_{k},t\right)\left(\frac{Y_{i}\left(Z_{k},t\right)}{\sum_{i=1}^{nc}Y_{i}\left(Z_{k},t\right)}\right)\right]=R_{ki}^{s} \end{array}$$

(26) $$\begin{array}{cc} \tau_{y}\frac{dY_{i}\left(Z_{k},t\right)}{dt}= & -\sum_{j=1}^{nz+1}A_{kj}\;\cdot \;Y_{i}\left(Z_{j},t\right)-A_{k,0}\;\cdot \;{\mathrm{BC}}_{t}+\\ & +K_{i}\left[P\left(\frac{X_{i}\left(Z_{k},t\right)}{\sum_{i=1}^{nc}X_{i}\left(Z_{k},t\right)}\right)-p\left(Z_{k},t\right)\left(\frac{Y_{i}\left(Z_{k},t\right)}{\sum_{i=1}^{nc}Y_{i}\left(Z_{k},t\right)}\right)\right]=R_{ki}^{t} \end{array}$$

(27) $$\tau_{p}\frac{dp\left(Z_{k},t\right)}{dt}=+\sum_{j=0}^{nz}A_{kj}\;\cdot \;p\left(Z_{j},t\right)+A_{k,nz+1}\;\cdot \;{\mathrm{BC}}_{p}-K_{pt}\frac{\sum_{i=1}^{nc}Y_{i}\left(Z_{k},t\right)}{p\left(Z_{k},t\right)}=R_{k}^{p}$$

where $\tau_{x}$, $\tau_{y}$, and $\tau_{p}$ are positive dummy relaxation parameters ($\tau_{x},\tau_{y},\tau_{p}>0$) used to adjust the strength of the pseudotransient terms; in the simulations reported in this work, $\tau_{x}=\tau_{y}=\tau_{p}=1$; k=0,…,nz are the collocation points in the ODE system described in Equations (25) and (27); and k=1,…,nz+1 are the collocation points in the ODE system described in Equation (26).

Finally, the ODE system can be solved by the integration methods available in the *scipy* 1.16.1 library, in Python 3.13.2 [37]. This library provides a wide range of solvers, including explicit Runge–Kutta methods of order 2(3) (*RK23*), 4(5) (*RK45*) and 8 (*DOP853*); the implicit Runge–Kutta method of the Radau IIA family of order 5 (*Radau*); a multi-step variable-order implicit method (*BDF*, orders 1 to 5); and the adaptive Adams/BDF method (*LSODA*). All these solvers were tested in this work to evaluate their performance and robustness. Among them, the *RK45* method stood out by offering the best compromise between computational efficiency and accuracy. Therefore, all results discussed in this study were obtained using the *RK45* solver.

Once the ODE system has been solved, the solution of the original system can be calculated by interpolating the solution at the final steady-state points and rescaling the variables. For the countercurrent case, Equations (28)–(30) are used.

(28) $$\frac{N_{s}x_{i}}{\sum_{i=1}^{nc}{\left(N_{s}x_{i}\right)}_{f}}={\left(N_{s}x_{i}\right)}^{*}=X_{i}\left(Z,t\to \infty \right)\approx \sum_{j=0}^{nz+1}l_{j}\left(Z\right)X_{i}\left(Z_{j},t\to \infty \right)$$

(29) $$\frac{N_{t}y_{i}}{\sum_{i=1}^{nc}{\left(N_{s}x_{i}\right)}_{f}}={\left(N_{t}y_{i}\right)}^{*}=Y_{i}\left(Z,t\to \infty \right)\approx \sum_{j=0}^{nz+1}l_{j}\left(Z\right)Y_{i}\left(Z_{j},t\to \infty \right)$$

(30) $$\frac{P_{t}}{P_{sf}}=P_{t}^{*}=p\left(Z,t\to \infty \right)\approx \sum_{j=0}^{nz+1}l_{j}\left(Z\right)p\left(Z_{j},t\to \infty \right)$$

where $l_{j}$ is an interpolating Lagrange polynomial:

(31) $$l_{j}\left(Z\right)=\prod_{\begin{array}{c} m=0\\ m\neq j \end{array}}^{nz+1}\frac{Z-Z_{m}}{Z_{j}-Z_{m}}$$

## 3. Results and Discussion

### 3.1. Analysis of the Numerical Approaches

Some iterative methods applied to steady-state equations may generate unstable formulations of the original problem. The pseudotransient approach was developed to overcome this by introducing artificial time-dependent terms, which guide the system toward the steady state in a more stable manner. According to Fletcher [19], Newton’s method is valuable for solving steady flow problems due to its rapid quadratic convergence; however, its main drawback is that the radius of convergence decreases as the number of variables increases, making it highly sensitive to initial guesses.

In the pseudotransient method, a fictitious time derivative is added to the steady-state system, transforming it into an equivalent unsteady problem that is marched in time until the steady state is reached. This artificial time parameter acts as a relaxation factor: with large time steps, the method behaves similarly to Newton’s method, taking aggressive steps toward reducing the residuals of the algebraic system; with small time steps, it resembles a relaxation scheme, slowly damping the residuals and ensuring stability [38].

This time-stepping mechanism improves the conditioning of the augmented Jacobian matrix, as the diagonal terms become more dominant, thereby enhancing stability and ensuring convergence even with poor initial estimates. For this reason, careful selection (and adaptive control) of the time-step size is crucial to minimize the number of iterations required until convergence.

As noted by Fletcher [19], many flow problems are governed by systems of partial differential equations, where iterative techniques such as multigrid methods are also applicable. In viscous problems with high Reynolds numbers, the system matrix tends to be poorly conditioned, making Newton’s convergence particularly difficult. Historically, Newton’s method has been more often applied in finite element formulations, while the pseudotransient approach has been widely adopted in finite difference methods [19,39]. Nonetheless, both are effective and complementary strategies for steady flow computations.

A case with extreme conditions was tested to analyze the two approaches and verify the numerical instabilities of their solutions. The mathematically extreme condition for the model occurs when a component has low concentration in the feed and, simultaneously, high permeability in the membrane. As a result, the physically feasible solution is close to other physically unfeasible solutions, making convergence with Newton’s method difficult, which has rapid convergence but is highly dependent on the initial guess, which can lead to unfeasible solutions. On the other hand, the pseudotransient method, with its temporal relaxation step, can reach the physically feasible solution more slowly; however, by conditioning the augmented Jacobian matrix, the integrator can move toward the feasible solution more prudently, reaching the desired solution, despite the extreme condition and the initial guess. Table 1 illustrates the base configuration of the simulated case for the analysis of the two solution approaches. In this case, the component with low concentration and high permeability is water ($H_{2}O$).

#### 3.1.1. Analysis of the Initial Random Estimate

An important comparison criterion corresponds to the convergence capacity of both approaches. As mentioned, the steady-state approach depends on the initial guess of the concentration profile along the membrane, which can be numerically restricted as a function of the boundary conditions of the problem. On the other hand, the initial guess of the concentration profile is considered as the initial condition for the integration problem of the pseudotransient approach.

To evaluate the convergence capacity of both methods, the scenario of the base case was considered under conditions of high permeance and low concentration of the water component, described in Table 1. The membrane $H_{2}O$ concentration profiles obtained using both numerical approaches (steady-state and pseudotransient), considering a random initial guess for the entire concentration profile, highlight the influence of the initial conditions on the computed solutions when a uniform random probability function is used to define both the profile and boundary setup (see Supplementary Material, Figures S1–S4).

The results show that the solution obtained with Newton’s method exhibits a divergent behavior for the $H_{2}O$ concentration profile, whereas the pseudotransient approach yields a smooth and convergent profile. A similar trend is observed for the membrane fiber side (tube side).

Particularly, defining the concentration profile as a random function corresponds to an extreme scenario since the boundary conditions of the problem are known, indicating the order of magnitude of the profiles in the tube and shell domains of the membrane. However, this type of analysis allows us to evaluate the advantages of using the pseudotransient approach for the numerical resolution of the phenomenological model in the present work, as it is very resilient to uncertainties in the initial guesses.

Although the pseudotransient approach showed a more robust behavior against adverse initialization conditions, the tube-side profile of the critical compound ($H_{2}O$) at very low concentrations exhibited a strong dependence on initial conditions. Additionally, random initialization can lead to longer transient trajectories, which can complicate the proper steady-state identification.

#### 3.1.2. Analysis of Permeances, Collocation Points, and Initial Estimates

This analysis compares four solver configurations: steady state (SS; Newton–Raphson, NR) and pseudotransient (PT; Runge–Kutta of order 4/5, RK45), each combined with either a *constant* or *linear* initial estimate. Results are assessed using accuracy metrics, mass-conservation checks, and computational performance.

**Figure 2:**
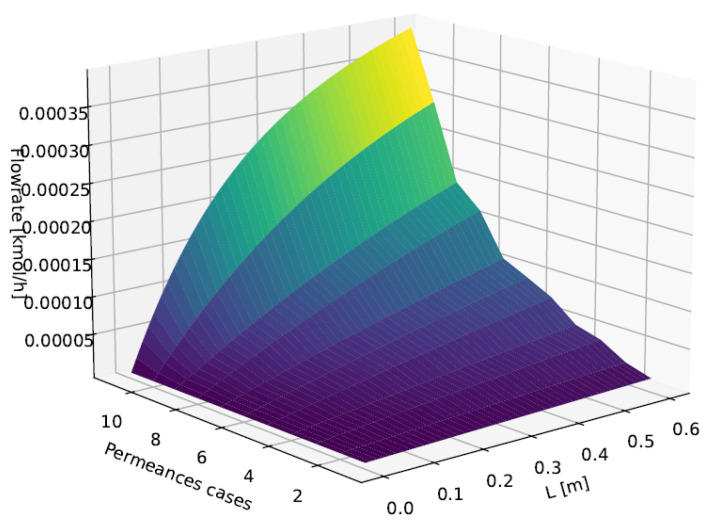
Reference flow rate profiles: water flow rate inside the fiber. The color gradient indicates the magnitude of the water flow rate, from lower values (dark colors) to higher values (bright colors).

A *base case* was generated for the sensitivity analysis with seven components in a co-current arrangement (Table 2 and Table 3). The sensitivity study considered simultaneous variations of permeances across 11 cases. In addition, we evaluated the impact of the total number of collocation points along the non-dimensional domain z∈[0,1], adopting n=3,4,5,6,7,8,9,10,16, and 21. It is important to emphasize that this benchmark does not aim to establish absolute accuracy against an external high-fidelity reference (e.g., a very fine BVP/FDM solution). Instead, the objective is to identify a practical trade-off among numerical robustness, computational performance, and solution accuracy for real-time and in-line applicability. Within this scope, the *SS-Linear* model with n=25 is used only as a high-resolution numerical reference for the component-wise comparison metric, whereas global and local mass-conservation metrics are evaluated independently from the governing conservation relations. As an example, Figure 2 shows the water flow rate profile inside the fiber for the reference configuration.

Given the large number of generated flow rate surfaces—combinations of 2 methods [SS(NR) and PT(RK45)], 2 initial estimates (constant and linear), 10 values of *n*, 2 streams (shell and tube), and 7 components, totaling 2×2×10×2×7=560 surfaces—Gauss–Legendre quadrature-based metrics were implemented. Mass balances were also applied directly at the collocation nodes, and computational performance was evaluated. These metrics prioritize mass conservation and computational efficiency. The main parameters and dimensions considered were as follows:

- c∈{0,1,…,10}: permeance cases ($n_{\mathrm{cases}}=11$);
- v∈{0,1,…,13}: component/variable index ($n_{\mathrm{vars}}=14$);
- *n*: total number of collocation points ($n=n_{i}+1$).

The metrics were defined as described in the following paragraphs.

**(i)**  **Global Mass Balance:**

At steady state without sweep, R(z)+P(z)=F; over the physical domain [0,L],

(32) $$\int_{0}^{L}\left[R\left(z\right)+P\left(z\right)\right]\;dz=F\;L$$

On the normalized domain [0,1], the integral can be approximated in the form:

(33) $$\int_{0}^{L}\left[R+P\right]\;dz\approx L\sum_{i=1}^{n_{i}}w_{i}\;\left[R\left(z_{i}\right)+P\left(z_{i}\right)\right]$$

where $z_{i}$ are the collocation nodes (roots of the Legendre polynomial), $n_{i}$ is the number of internal collocation nodes, and $w_{i}$ are the Gauss–Legendre quadrature weights, exact for any polynomial up to degree $2n_{i}-1$. These weights satisfy $\sum_{i}w_{i}=1$ on [0,1], ensuring that the integral of a constant reproduces FL exactly.

Thus, for each collocation size *n*, permeance case *c*, and variable *v* (7 components), the mean relative global mass-balance error is:

(34) $$\varepsilon_{n,c}^{\left(\mathrm{GMB}\right)}\;\left[\%\right]=100\;\frac{\left|\;L\sum_{v=1}^{7}\sum_{i=1}^{n-1}w_{i}\;\left[R_{c,v}\left(z_{i}\right)+P_{c,v}\left(z_{i}\right)\right]-F\;L\;\right|}{F\;L}$$

(35) $$\varepsilon_{n}^{\left(\mathrm{GMB}\right)}\;\left[\%\right]={\mathrm{mean}}_{c}\;\left[\varepsilon_{n,c}^{\left(\mathrm{GMB}\right)}\right]$$

**(ii)**  **Computational performance (speedup):**

Performance gain is defined with respect to a common time reference $t_{\mathrm{ref}}$

(36) $${\mathrm{Speedup}}_{n}=\frac{t_{\mathrm{ref}}}{t_{n}}$$

adopting as reference the slowest case, *PT + Constant* with n=21 (300.057 s), which represents the wall time to run all permeance cases for a given *n*.

**(iii)**  **Pointwise conservation (local residual):**

For each *n*, *c*, and *v*, pointwise mass closure at each collocation node is:

(37) $${\mathrm{Res}}_{n,c}\left(z_{i}\right)=\left|\sum_{v=1}^{7}\left[R_{c,v}\left(z_{i}\right)+P_{c,v}\left(z_{i}\right)\right]-F\right|$$

The worst-case residual is:

(38) $${\mathrm{Res}}_{n}^{\max}=\max_{c,i}\;{\mathrm{Res}}_{n,c}\left(z_{i}\right)$$

**(iv)**  **Component-wise analysis:**

Each component/stream integral is compared against a high-accuracy reference (*SS + Linear*, n=25). The choice of this high-resolution setting is restricted to this specific metric and is supported by mesh-refinement trends, which indicate progressive convergence as *n* increases, together with smooth profiles and no visible numerical artifacts at n=25. Therefore, this configuration is adopted only as a numerical reference for comparison, and not as a recommended operational setting for real-time applications. For each permeance case *c* and variable *v* (7 components × 2 streams), one can define:

(39) $$A_{n,c,v}=L\sum_{i=1}^{n-1}w_{i}\;N_{c,v}\left(z_{i}\right)$$

(40) $$A_{c,v}^{\mathrm{ref}}=A_{n=25,c,v}^{\mathrm{SS}\;+\;\mathrm{Linear}}$$

The aggregated mean error:

(41) $$\varepsilon_{n}^{\left(\mathrm{cw}\right)}=\frac{1}{154}\sum_{c=0}^{10}\left[\sum_{v=0}^{6}\left|\frac{A_{c,v}^{\mathrm{tube}}-A_{c,v}^{\mathrm{ref},\mathrm{tube}}}{A_{c,v}^{\mathrm{ref},\mathrm{tube}}}\right|+\sum_{v=0}^{6}\left|\frac{A_{c,v}^{\mathrm{shell}}-A_{c,v}^{\mathrm{ref},\mathrm{shell}}}{A_{c,v}^{\mathrm{ref},\mathrm{shell}}}\right|\right]\times 100\%$$

Here, $A_{c,v}^{\mathrm{ref}}$ is the reference area and $N_{c,v}\left(z_{i}\right)$ denotes the local flow rate for case *c* and variable *v* (7 components in *R* and *P*) at node $z_{i}$.

This metric provides a granular view of convergence, enabling separate assessment of each component and stream. It allows one to pinpoint species with problematic convergence, validate uniform accuracy across components, and detect localized numerical instabilities that global metrics may conceal. In total, 154 individual items are evaluated (11 permeance cases × 14 variables across components and streams), offering the most detailed assessment of numerical accuracy among the tested configurations.

**Figure 3:**
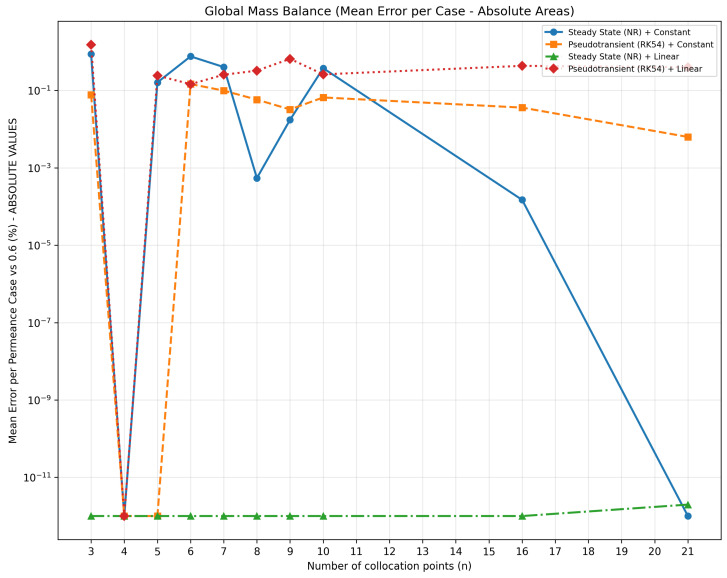
Global mass-balance analysis.

The comparative assessment is consolidated by four key plots that fully characterize the numerical behavior of the configurations. Figure 3 quantifies global mass-balance accuracy, revealing physical errors on the order of $10^{-14}$% for *SS + Linear* and identifying for the base-case sensitivity dataset the critical threshold n=4 at which all configurations achieve satisfactory convergence under the metrics defined in this subsection.

**Figure 4:**
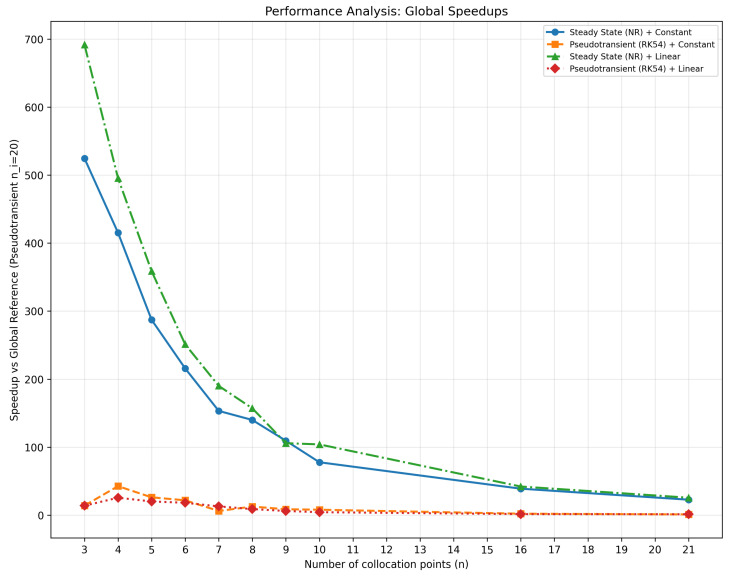
Performance analysis.

Figure 4 establishes computational efficiency benchmarks, showing that *SS + Linear* attains speedups up to 692×, turning minute-long simulations into fractions of a second.

**Figure 5:**
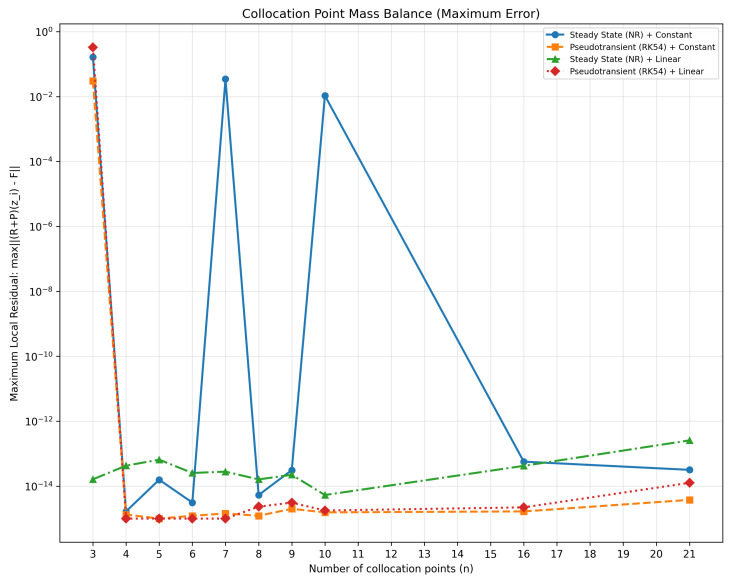
Collocation node mass-balance analysis.

Figure 5 validates pointwise mass conservation at each collocation node, with *SS + Linear* maintaining local residuals at machine precision (∼10^−13^ kmol/h) and revealing significant instabilities in pseudotransient configurations.

**Figure 6:**
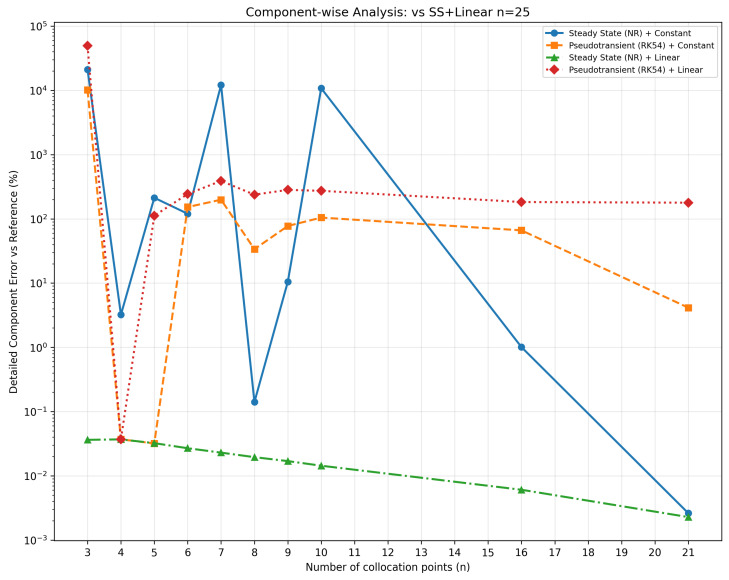
Component-wise analysis.

Finally, Figure 6 provides the most detailed assessment via the granular comparison of 154 individual items against the *SS + Linear* n=25 reference, detecting component-specific deviations that could be masked by global metrics and confirming that within the base-case sensitivity study n≥4 ensures uniform convergence across the molecular spectrum. Together, these plots establish *SS + Linear* with n=4 as the optimal configuration for this base-case scope, combining machine-precision accuracy, complete physical consistency, and superior computational efficiency.

**Figure 7:**
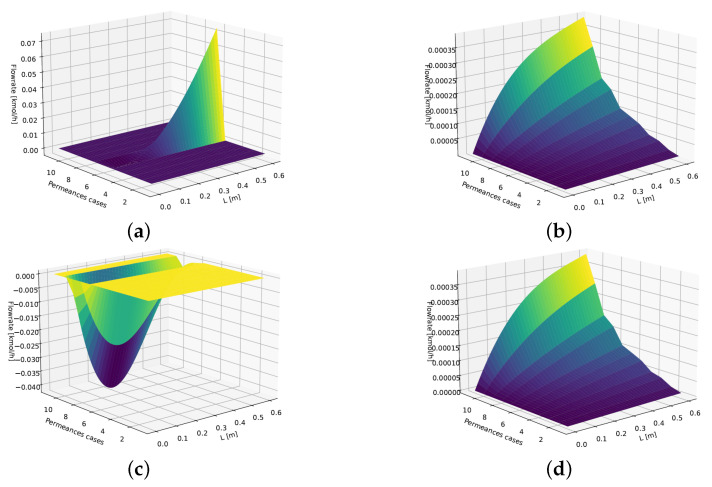
Sensitivity analysis: $H_{2}O$ flow rate inside the fiber—worst and best cases. The color gradient indicates the magnitude and sign of the flow rate, with darker colors representing lower values, brighter colors representing higher positive values, and the negative region shown in panel (**c**). (**a**) Pseudotransient (RK45)—linear—n=3. (**b**) Pseudotransient (RK45)—constant—n=4. (**c**) Steady state (NR)—constant—n=3. (**d**) Steady state (NR)—linear—n=3.

Figure 7 illustrates the best and worst cases (*SS + Constant*, *PT + Constant*, *SS + Linear*, and *PT + Linear*), exemplified by the water flow rate inside the fiber—the critical variable where most gross flow rate errors (e.g., negative values) occur.

When using a constant initial estimate, the *PT + Constant* approach produced more stable solutions and was closer to the reference than *SS + Constant*, outperforming it in 6 of the 10 collocation-size cases according to the global mass-balance metric and in 100% of the cases in the nodewise residual analysis.

The best results for both constant and linear initial estimates occurred with four collocation points, whereas the worst performance was observed with three points, likely due to the symmetry of the interpolating polynomial, which fails to represent the inherently asymmetric flow rate profile. Thus, n=4 represents the optimal trade-off between accuracy and computational cost for the base-case sensitivity analysis and for the metrics considered in this subsection.

A linear initial estimate strongly favored the SS(NR) method, which reached machine precision with near-immediate convergence. In contrast, the same estimate hindered the PT(RK45) model, whose robustness is more evident when the initial profile is constant.

Therefore, when the solution profile is known, *SS + Linear* is the most efficient and accurate option; when it is unknown, *PT + Constant* offers greater robustness. Overall, within the base-case sensitivity analysis, the optimal configuration is *SS* + *Linear* + *n* = 4, balancing accuracy, stability, and computational performance; however, the dedicated mesh-convergence validation required finer meshes, with convergence at 12 collocation points for Case 1 and 10 collocation points for Case 2 (Section 3.2.1).

Table 4 summarizes the key metrics for the four configurations. Among them, *SS + Linear* stands out as the only configuration that is physically consistent in all cases, achieving guaranteed convergence, zero physical error, and speedups up to 692× relative to the baseline.

The algorithm also detected physical inconsistencies—notably negative flow rates—causing mass-balance masking effects in 75% of the configurations. In particular, *PT + Linear* exhibited up to ten negative values per case, whereas *SS + Constant* showed masking effects of up to 2.4%. Only *SS + Linear* maintained 100% physical consistency and perfect mass balance for the tested base-case configurations.

From a performance standpoint, within the base-case sensitivity scenarios, *SS + Linear* with n=3 to 4 achieved speedups of 700–500×, immediate convergence, and zero physical error, making it a strong candidate for industrial applications. For cross-validation and more sensitive cases, *PT + Constant* (n≥4) is recommended due to its higher robustness, albeit at roughly 20× the computational cost; in addition, mesh-refinement studies may require 10–12 collocation points depending on the case. For online monitoring applications, however, configuration selection is preferably weighted toward computational performance once convergence and physical consistency are met, since small internal-fiber profile deviations can be compensated by real-time permeance estimation and are typically negligible compared with measurement uncertainty in real-process data. The combinations *SS + Constant* and *PT + Linear* showed inconsistent behavior and are not recommended.

In practical terms, for the investigated benchmark space, efficiency gains were substantial: simulations that previously required minutes now complete in fractions of a second, with 100% physical consistency, 0% global mass-balance error, and no observed convergence failures. This improvement enables extensive parametric studies, permeance optimization, and feasibility analyses with high numerical reliability.

Therefore, for the base-case sensitivity scenario, the *SS + Linear* (n=4) configuration is the optimal solution for hollow-fiber membrane simulations via orthogonal collocation, combining:

- Mathematical precision: physical error =0.0000%;
- Complete physical consistency: 100% positive flow rates;
- Computational efficiency: up to 500× faster;
- Numerical robustness: robust convergence for the tested cases.

### 3.2. Mass Balance

#### 3.2.1. Mesh Convergence Analysis

Mesh convergence analysis is an important step in orthogonal collocation, as it involves increasing the number of collocation points and analyzing the impact of this process on the accuracy of the solution. The evaluation of the convergence of the iterative process is carried out by computing the error (ε) of the variable *x* at iteration *k*, which must be smaller than the tolerance (δ) herein specified as 5 %, following the relation $\varepsilon =\frac{|x^{\left(k\right)}-x^{*}|}{x^{*}}\leq \delta$, where $x^{*}$ is the known reference value of the variable.

The mesh convergence analysis employed in this study is illustrated through the simulation of Cases 1 and 2 taken from Helmersen [23] presented in Table 5, using the steady-state approach. The reference variables considered were as follows: (i) Case 1: fiber pressure and flow rates of ${\mathrm{CO}}_{2}$ and $H_{2}O$; and (ii) Case 2: fiber pressure and flow rates of ${\mathrm{CO}}_{2}$ and ${\mathrm{CH}}_{4}$. In both cases, the operation is carried out in a countercurrent flow, with the feed entering on the shell side at the point corresponding to z=1, as shown in Figure 1.

- ***Case 1***

**Figure 8:**
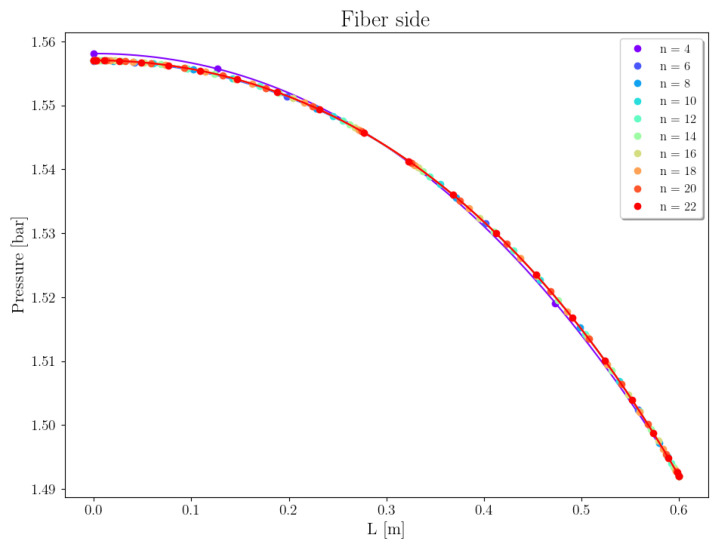
Mesh convergence analysis of pressure on fiber side, Case 1.

Case 1 concerns the separation of ${\mathrm{CO}}_{2}$ from the gas mixture with a composition as shown in Table 6, using a PI-Carbon membrane. Figure 8 depicts the pressure profiles within the tube along the fiber length, varying the number of collocation points from 4 to 22. It can be observed that as the number of points increases from 4 to 6, the curves shift slightly in the direction of reducing the fiber pressure at z=0, which drops by approximately 0.001 bar. Since this variation has a low value, the relative error obtained is reduced, even when using only 6 collocation points ($\varepsilon_{P_{t}}=0.06\%$).

**Figure 9:**
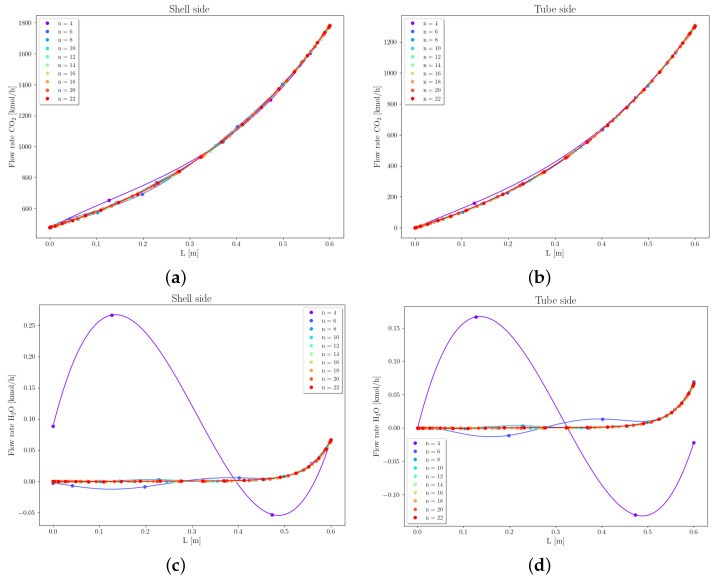
Mesh convergence analysis for the flow rate of ${\mathrm{CO}}_{2}$ and $H_{2}O$, Case 1. (**a**) ${\mathrm{CO}}_{2}$, shell. (**b**) ${\mathrm{CO}}_{2}$, fiber. (**c**) $H_{2}O$, shell. (**d**) $H_{2}O$, fiber.

The mesh convergence analysis for the flow rate of ${\mathrm{CH}}_{4}$, $C_{2}H_{6}$, $C_{3}H_{8}$, $C_{4}H_{10}$, and $C_{5+}$ (Figures S5–S7 on Supplementary Material) shows that the change in the number of points did not significantly impact the hydrocarbon flow profiles, both inside and outside the fiber. This is because the hydrocarbon flows along the fiber exhibit linear profiles, which can be accurately represented using a small number of collocation points. Conversely, the flow rates of ${\mathrm{CO}}_{2}$ and $H_{2}O$ exhibit curved profiles along the fiber length due to their higher permeances. In particular, the flow rate of $H_{2}O$ shows pronounced oscillatory behavior when a limited number of collocation points is used. Therefore, mesh refinement directly affects the response of these variables, as shown in Figure 9, underscoring the importance of performing mesh convergence analysis when employing the orthogonal collocation method. The calculated relative errors indicate that the solution of the system of differential equations converges when employing 12 collocation points.

- ***Case 2***

**Figure 10:**
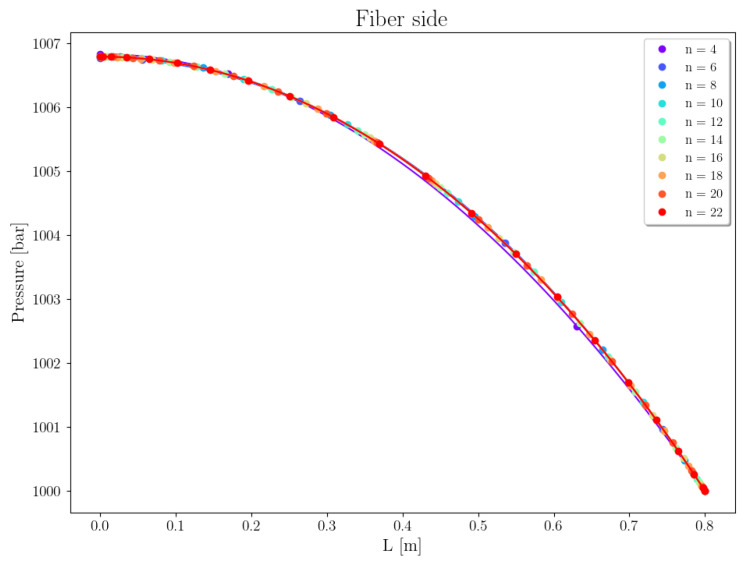
Mesh convergence analysis of pressure on the fiber side, Case 2.

Case 2 concerns the separation of the gas mixture with molar composition of $x_{{\mathrm{CO}}_{2}}=10\%$ and $x_{{\mathrm{CH}}_{4}}=90\%$, using a CA-Carbon membrane. The permeances considered are $P_{{\mathrm{CO}}_{2}}=1.749\times 10^{-9}$ [mol/m^2^ Pa s] and $P_{{\mathrm{CH}}_{4}}=1.227\times 10^{-10}$ [mol/m^2^ Pa s]. Figure 10 shows the pressure profiles on the fiber side, varying the number of collocation points. It can be observed that increasing the number of collocation points causes slight curvature shifts in the profiles. The relative error at z=0 remained consistently low, even with only 8 collocation points ($\varepsilon_{P_{t}}$ = 0.002%).

**Figure 11:**
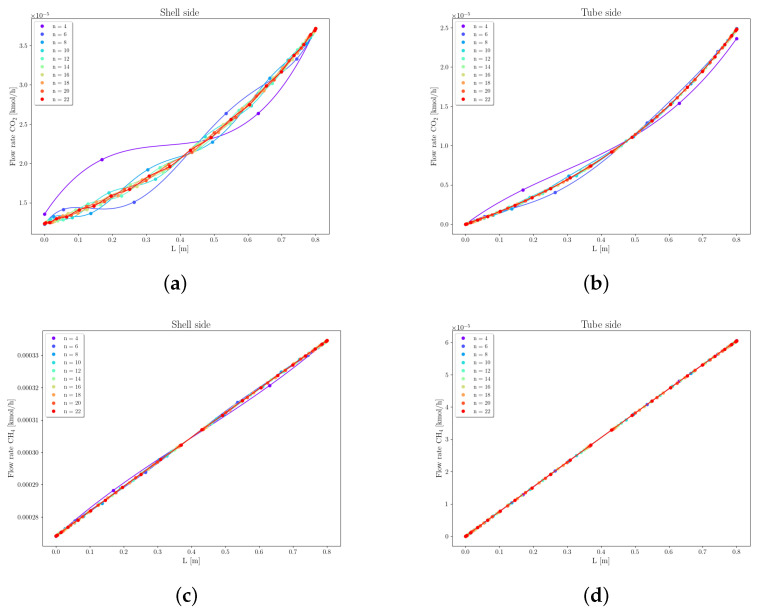
Mesh convergence analysis of flow rate of ${\mathrm{CO}}_{2}$ and ${\mathrm{CH}}_{4}$, Case 2. (**a**) ${\mathrm{CO}}_{2}$, shell. (**b**) ${\mathrm{CO}}_{2}$, fiber. (**c**) ${\mathrm{CH}}_{4}$, shell. (**d**) ${\mathrm{CH}}_{4}$, fiber.

With respect to the component flow rates, Figure 11 shows that the number of collocation points does not impact the flow profiles of ${\mathrm{CH}}_{4}$ on the fiber side (tube side). However, on the shell side, a slight variation in the flow profiles of ${\mathrm{CH}}_{4}$ is observed as the number of collocation points increases. These profiles are linear and, therefore, behave similarly to the hydrocarbons in Case 1, being minimally influenced by mesh refinement. In contrast, the flow profiles of ${\mathrm{CO}}_{2}$ along the membrane, on both shell and fiber sides, are strongly influenced by the number of collocation points. This is because this component exhibits higher permeation, leading to curvatures in its flow profiles.

It is important to note that the ${\mathrm{CO}}_{2}$ flow profiles on the shell side exhibit oscillations that persist even with increased points. This behavior was also observed in the profiles presented by [23], and it is inherent to the orthogonal collocation method. The calculated relative errors indicate that the solution of the system of differential equations reaches convergence when using 10 collocation points.

#### 3.2.2. Mass-Balance Validation

The proposed mass balance was validated by comparing the predicted values with experimental data obtained by Pan [9]. In their study, multicomponent gas separation was conducted using an asymmetric cellulose acetate hollow-fiber membrane. The membrane employed was a miniature fiber module consisting of 36 cm long fibers arranged in a U-loop configuration, resulting in a total of $n_{fb}=20$ fibers. The outer and inner diameters of these fibers were $d_{o}$ = 200 µm and $d_{i}$ = 80 µm, respectively. The high-pressure feed gas was introduced into the shell side of the module at a controlled temperature and pressure, while the permeate was withdrawn from the tube side using concurrent and countercurrent flow patterns. This study focused on the separation of $H_{2}$ from ammonia plant purge gas and the separation of ${\mathrm{CO}}_{2}$ and $H_{2}S$ from sour gas mixtures.

Given that the primary focus of this study is to establish a model for characterizing the separation of ${\mathrm{CO}}_{2}$ and $H_{2}S$ from natural gas utilizing membrane technology, a validation study using experimental data from the work of Pan [9] was conducted, which employed a gas mixture with compositions that were similar to the ones observed in Brazilian oil wells. The experimental conditions are detailed in Table 7, with the specific case denoted as Case 3. The system operates under isothermal conditions at 50 °C, employing a countercurrent flow pattern.

In addition to conducting experiments, Pan [9] proposed a model to describe gas permeation through the hollow-fiber membrane, from which they were able to estimate the selectivity of the components. However, it should be noted that the model proposed in this work makes different assumptions, demanding a new round of parameter estimation. First, the feed flow rate was estimated using the parameters provided by Pan [9] and by using the first experiment (with a stage-cut of approximately 3.5) as a reference. This procedure was necessary because the author did not provide flow rate data, only stage-cut data. As a result, a feed flow rate ($N_{f}$) of $1\times 10^{-4}$ mol/s was obtained, which was considered constant in all experiments. Subsequently, the estimation of the permeance of the gaseous components for all experiments was carried out. The selectivity of $N_{2}$ relative to ${\mathrm{CH}}_{4}$ was set to unity, in accordance with the findings of Pan [9]. Concurrently, the permeance values for ${\mathrm{CH}}_{4}$, ${\mathrm{CO}}_{2}$, and $H_{2}S$ were estimated to allow model fitting to the experimental dataset.

Accordingly, this validation should be interpreted as calibration followed by agreement: the feed flow rate is reconstructed from reported stage-cut information, and component permeances are re-estimated to match the experimental cases. Within this scope, the objective is to verify whether the coupled equations reproduce the observed trends consistently, rather than to claim fully predictive validation with a fixed *a priori* parameter set.

**Figure 12:**
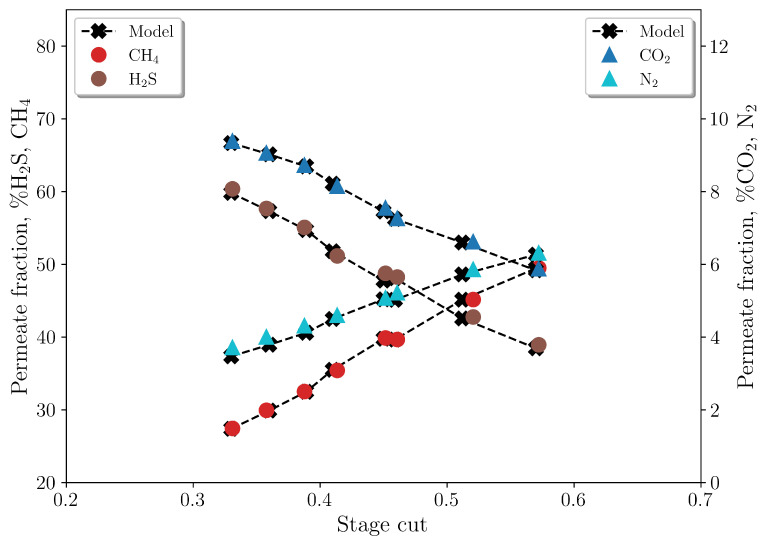
Comparison between model predictions and experimental data for Case 3.

**Figure 13:**
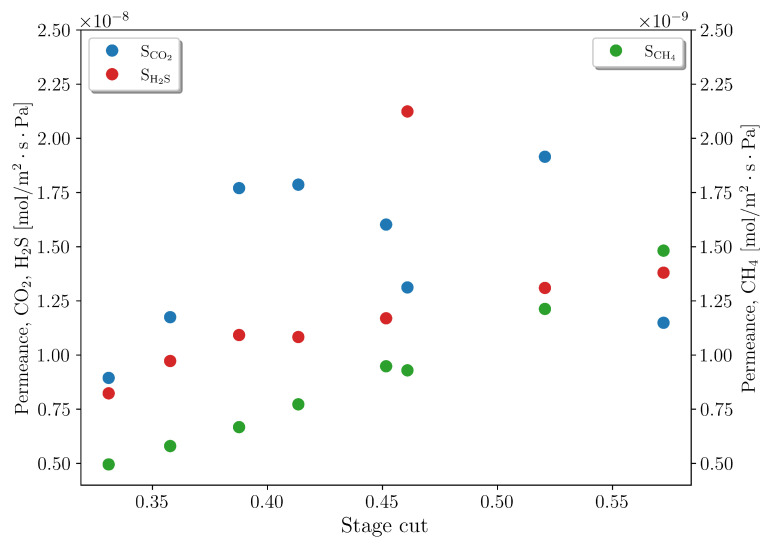
Estimated permeances for the different experimental conditions of Case 3.

Figure 12 presents the comparison between the model predictions and experimental data, illustrating the effectiveness of the proposed mass balance in accurately describing gas separation across various stage-cuts. Figure 13 shows the estimated permeances to represent the different experimental conditions. It can be observed that $P_{CO_{2}}$ and $P_{H_{2}S}$ are high and approximately equal in the various experiments, as observed by Pan [9]. On the other hand, the estimated $P_{CH_{4}}$ increases with the increase in the stage-cut, as a consequence of the rising concentration of this compound in the permeate stream.

### 3.3. Energy Balance

#### 3.3.1. Energy Balance Validation

The proposed energy balance was validated by comparing the predicted values with experimental data obtained by Lock et al. [40]. In their study, multicomponent gas separation was conducted using a commercial polyimide membrane hollow fiber with a porosity of 0.5. The membrane employed was a miniature fiber module consisting of an active fiber 28 cm in length, with outer and inner diameters of $d_{o}$ = 400 µm and $d_{i}$ = 180 µm, respectively. The high-pressure feed gas was introduced into the shell side of the module, while the permeate was withdrawn from the tube side using a countercurrent flow pattern. This study focused on the capture of ${\mathrm{CO}}_{2}$ from natural gas, represented by a mixture of ${\mathrm{CO}}_{2}$, ${\mathrm{CH}}_{4}$ and $C_{5}H_{12}$. The experimental conditions are presented in Table 8.

In addition to conducting experiments, Lock et al. [40] proposed a model to describe gas permeation through the hollow-fiber membrane, considering the Joule–Thomson thermodynamic effect on temperature and membrane permeance in a two-dimensional radial crossflow mechanism with variation along both axial and radial directions. Using the proposed model, they were able to estimate the temperature change. However, since the model proposed in the present work is based on different assumptions, a new round of parameter estimation is necessary to determine the global heat transfer coefficient. A set of experiments was used to perform the parameter estimation procedure, using the experimental permeate-stream temperature as the reference, as shown in Table 9.

Comparing the model predictions and experimental data presented in Table 9, it is possible to conclude that the proposed energy balance accurately describes gas separation at different feed compositions. The estimated global heat transfer coefficient value is different for each experimental condition. The model estimates a higher *U* value as the ${\mathrm{CO}}_{2}$ concentration increases, since under these conditions, the Joule–Thomson effect is more significant, leading to a larger decrease in permeate temperature. Therefore, to achieve the experimentally observed maximum temperature drop of 4 °C, an increase in heat exchange between the retentate and permeate streams is estimated.

In this context, the global heat transfer coefficient is treated as an effective parameter estimated separately for each experiment, rather than as a universal constant. Therefore, the energy-balance assessment is interpreted as calibration followed by agreement, which demonstrates model flexibility and consistency across operating conditions without claiming parameter universality.

The simulated data obtained by Coker et al. [26] were used here to validate the proposed energy balance for describing the separation of ${\mathrm{CO}}_{2}$ from binary and multicomponent mixtures using an industrial-scale polyimide hollow-fiber membrane module. The authors developed a simulator for hollow-fiber membrane modules that incorporated coupled mass and energy balances, which were solved using a finite difference computational scheme. The simulations were performed for a membrane module on an industrial scale, operating in a countercurrent pattern, where the feed gas was introduced into the shell side, while the permeate flowed on the tube side, as shown in Table 10. The simulations focused on the separation of ${\mathrm{CO}}_{2}$ from natural gas and from ${\mathrm{CH}}_{4}$, varying the initial concentration of ${\mathrm{CO}}_{2}$ and the feed flow. The simulated conditions are presented in Table 10.

**Figure 14:**
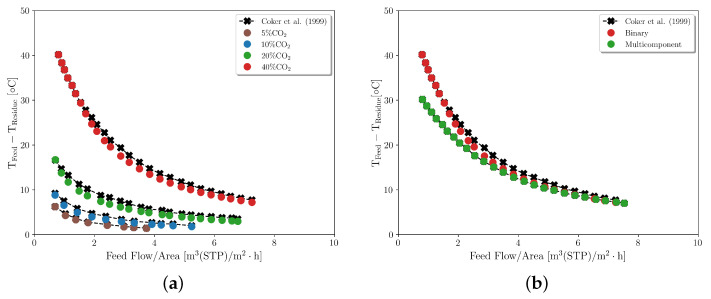
Model validation with simulated data from Coker et al. [26]: (**a**) binary mixture of ${\mathrm{CO}}_{2}/{\mathrm{CH}}_{4}$; (**b**) binary mixture is 40% ${\mathrm{CO}}_{2}$ and 60% ${\mathrm{CH}}_{4}$. Multicomponent mixture contains 40% ${\mathrm{CO}}_{2}$, 55.89% ${\mathrm{CH}}_{4}$, 1.72% $N_{2}$, 1.74% $C_{2}H_{6}$, and 0.65% $C_{3}H_{8}$.

The model proposed by Coker et al. [26] is based on assumptions that differ from those adopted in the present framework. Both approaches consider steady-state one-dimensional plug flow, negligible axial mixing, and pressure drop in the fiber lumen, while shell-side pressure variation is neglected. However, the present model includes a different thermal closure, explicitly coupling shell- and tube-side energy balances and Joule–Thomson contributions within the adopted constitutive and numerical framework. In addition, the present implementation uses an orthogonal-collocation formulation with a dedicated treatment of the energy-balance boundary condition when permeate flow approaches zero, whereas Coker et al. [26] used a finite-difference strategy. Because of these structural differences, thermal parameters are not directly transferable between models. Therefore, a new parameter-estimation round was required, using the residue temperatures reported for different feed flows as references. Observing Figure 14, it is possible to conclude that the proposed energy balance accurately describes gas separation at different feed flows and feed compositions, with an estimated global heat transfer coefficient in the range of 2–10 $W\;\cdot \;m^{-2}\;\cdot \;K^{-1}$ for the simulated conditions.

#### 3.3.2. Computational Cost Analysis

Another relevant point concerns the computational cost of performing each numerical approach. As mentioned in the preceding section, the steady-state approach involves solving a nonlinear algebraic system of equations, while the pseudotransient approach entails solving a set of ordinary differential equations.

To compare both approaches, tests were conducted to measure the CPU time for different configuration scenarios of the cases defined in this study. These tests were carried out on a 2.60 GHz CPU with a standard setup.

**Figure 15:**
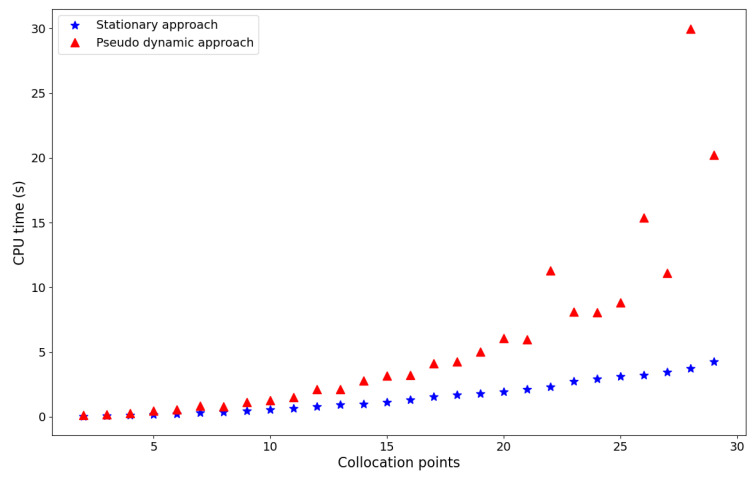
Comparison of the numerical approaches in terms of computational cost (Case 1).

Figure 15 depicts the CPU time behavior required by both methodologies to solve Case 1 as the number of collocation points varies. As observed, the pseudotransient approach proved to be more computationally demanding than the steady-state approach, and both methodologies exhibited similar behavior for a low number of collocation points.

As expected, the computational cost of the set of differential equations increased with the number of collocation points since the size of the system became greater. Furthermore, the size of the temporal vector and the initial conditions used during the integration affect the performance of the pseudotransient approach.

It is worth mentioning that the above case involved solving only the mass-balance equations. Therefore, to evaluate the boundary-condition treatment proposed in the previous sections and its effects on computational performance, Case 2 was considered, with the times required to solve the mass-balance and energy-balance equations measured separately.

**Figure 16:**
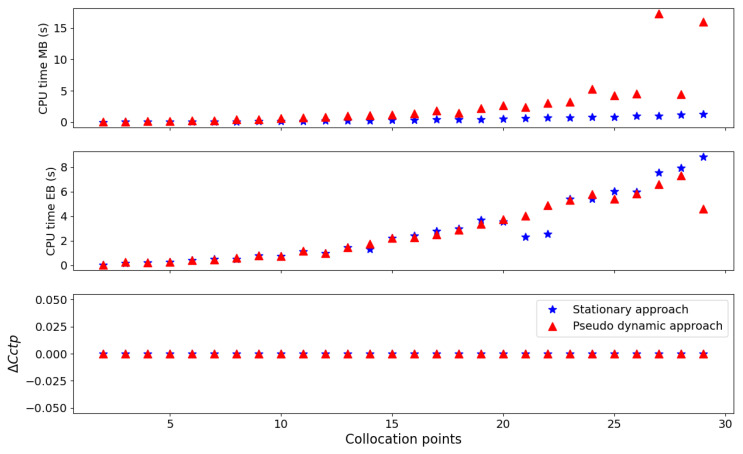
Comparison of the numerical approaches in terms of computational cost (Case 2).

Figure 16 illustrates the performance comparison of both methodologies. Regarding the solution of the mass-balance equation, the steady-state approach exhibited better performance. However, when considering the energy-balance equations, the pseudotransient approach prevailed in scenarios with a high number of collocation points. Figure 16 also depicts the behavior of the metric $\Delta C_{ctp}=\left|T_{t}^{*}\left(z^{*}=0\right)-\frac{T_{t0}}{T_{f}}\right|$, which is dimensionless [-] and represents the residual mismatch of the treated temperature boundary condition at the permeate inlet. Therefore, values close to zero indicate that the boundary-condition constraint in Equation (18) is satisfied.

From an industrial real-time perspective, previous studies with spiral-wound membranes reported full-unit simulation times of approximately 13 s and convergence of parameter-estimation procedures within a 5-min sampling interval [24,41]. In the present hollow-fiber framework, the best-performing numerical setup is approximately 500 times faster than the most computationally demanding scenario (about 300 s), corresponding to an average computational time of approximately 0.055 s per membrane module. Considering a representative arrangement with up to 12 membrane elements in series per stage, this leads to an estimated 0.66 s per stage and approximately 1.3 s for a two-stage unit. This estimated computational demand is about one order of magnitude lower than previously reported full-unit times, supporting the computational suitability of the proposed framework for real-time monitoring-oriented applications.

## 4. Conclusions

In this work, a comprehensive phenomenological and numerical framework was developed and analyzed for the simulation of hollow-fiber membrane modules, with particular emphasis on the solver behavior, numerical robustness, and computational efficiency, aiming at the in-line and real-time implementation of the model. The governing equations, including coupled mass, momentum (through pressure drop), and energy balances, were discretized using a rigorous orthogonal collocation formulation and solved using both steady-state Newton–Raphson and pseudotransient approaches. Particularly, an original and consistent numerical treatment was proposed to address the boundary condition of the energy balance at locations where the permeate flow vanishes.

The obtained results clearly showed that the steady-state Newton–Raphson method, when combined with physically consistent initial profiles, provided the best overall numerical performance. In particular, for the base-case sensitivity analysis, the use of a linear initial guess and a mesh consisting of four orthogonal collocation points consistently yielded the fastest convergence while maintaining numerical robustness and physical consistency across a wide range of operating conditions, as required by real-time implementations. However, the pseudotransient formulation was shown to be particularly useful in extreme regimes involving high-permeance components at low concentrations and flow rates, such as $H_{2}O$ and ${\mathrm{CO}}_{2}$ encountered in acid-gas removal processes. In these cases, the pseudotransient approach provided reliable convergence when steady-state solvers became sensitive to initialization, serving as an effective auxiliary or initialization strategy.

The model was validated against experimental data from the literature for both mass and energy balances, and extensive sensitivity and mesh convergence analyses were conducted. These analyses confirmed that components with higher permeance and strongly nonlinear profiles require finer spatial discretization to achieve convergence. The original approach used to describe the boundary condition of the energy balance enabled the stable incorporation of thermal effects and the Joule–Thomson phenomenon along the membrane length.

Overall, the obtained results indicate that the recommended default numerical configuration for screening and real-time studies consists of a mesh containing four orthogonal collocation points combined with a steady-state Newton–Raphson solver and a linear initial guess approach. Nevertheless, mesh-convergence validation showed that the optimal collocation size is case-dependent, with convergence reached at 12 points in Case 1 and 10 points in Case 2. For monitoring applications, the final numerical choice should be primarily oriented toward computational performance rather than maximum mesh-convergence accuracy, provided that physical consistency is preserved. In this regime, the small discrepancies observed in internal-fiber profiles can be continuously corrected through real-time permeance estimation and are negligible relative to the uncertainty of measured data in real industrial processes. Within the scope of the present study, this configuration is computationally suitable for real-time simulation and monitoring-oriented applications. Nevertheless, full deployment in digital twins, online parameter updating, reduced-order coupling, and closed-loop control was not demonstrated here and remains for future work.

## Figures and Tables

**Table 1:** Base case settings for numerical analysis.

Items	
Configuration	Co-current
Number of components	7
Constant pressure (tube side)	Yes
Sweep Gas	No
Newton’s method solver	*fsolve* from *scipy.optimize*
Pseudotransient method solver	Yes (*solve_ivp* [RK45] from *scipy.integrate*)
Components	${\mathrm{CO}}_{2}$, $C_{1}$, $C_{2}$, $C_{3}$, $C_{4}$, $C_{5}$, and $H_{2}O$
$P_{i}$ [kmol/(m^2^ · h · kPa)]	$10^{-5}\times \left[35,4.5,0.5,0.05,0.015,0.005,95\right]$
$A_{T}$ [m^2^]	$\pi \;\cdot \;d_{o}\;\cdot \;L\;\cdot \;n_{fb}$
$d_{o}$ [m]	$2.5\;\cdot \;10^{-4}$
*L* [m]	0.6
$n_{fb}$	6000
${\left(N_{s}x_{i}\right)}_{f}$ [kmol/h]	$\left[8.98\;\cdot \;10^{-2},80.54\;\cdot \;10^{-2},6.43\;\cdot \;10^{-2},2.46\;\cdot \;10^{-2},1.52\;\cdot \;10^{-2},6.75\;\cdot \;10^{-4},3.35\;\cdot \;10^{-10}\right]$
${\left(N_{t}y_{i}\right)}_{p}$ [kmol/h]	[0,0,0,0,0,0,0]
$P_{s}$ [kPa]	3500
$P_{t}$ [kPa]	100

**Table 2:** Base case settings for sensitivity analysis.

Items	
Configuration	Co-current
Number of permeances	7
Number of collocation points	15
Constant pressure (tube side)	No
Sweep Gas	No
Newton’s method solver	Yes (*fsolve* from *scipy.optimize*)
Pseudotransient method solver	Yes (*solve_ivp* [RK45] from *scipy.integrate*)
Components (*i*)	${\mathrm{CO}}_{2}$, $C_{1}$, $C_{2}$, $C_{3}$, $C_{4}$, $C_{5}$, and $H_{2}O$
$P_{i}$ [kmol/(m^2^ · h · kPa)]	$10^{-5}\times \left[35,1.5,0.5,0.05,0.015,0.005,2.5\right]$
$d_{o}$ [m]	$2\;\cdot \;10^{-4}$
*L* [m]	0.6
*n_fb_*	2000
$A_{T}$ [m^2^]	$\pi \;\cdot \;d_{o}\;\cdot \;L\;\cdot \;n_{fb}$
${\left(N_{s}x_{i}\right)}_{f}$ [kmol/h]	$10^{-2}\times \left[25,65,6.5,1.95,1,0.5,0.05\right]$
${\left(N_{t}y_{i}\right)}_{p}$ [kmol/h]	[0,0,0,0,0,0,0]
$P_{s}$ [kPa]	3500
$P_{t}$ [kPa]	700

**Table 3:** Permeance parameters in the sensitivity analysis.

Cases	Permeances ($P_{i}$ ) [kmol/(m^2^ · h · kPa)]
1	$0.1\times P_{i}$
2	$0.2\times P_{i}$
3	$0.4\times P_{i}$
4	$0.5\times P_{i}$
5	$0.8\times P_{i}$
6 (base)	$1\times P_{i}$
7	$1.2\times P_{i}$
8	$2\times P_{i}$
9	$2.5\times P_{i}$
10	$5\times P_{i}$
11	$10\times P_{i}$

**Table 4:** Comparison of evaluated numerical configurations.

Configuration	Physical Issues	Max. Speedup	Max. Physical Error
**SS + Linear**	**0/10 cases**	**692×**	**0.0000%**
SS + Constant	8/10 cases	525×	1.53%
PT + Constant	8/10 cases	14×	0.11%
PT + Linear	9/10 cases	14×	1.37%

Boldface indicates the best results among the evaluated configurations in each column.

**Table 5:** Operational conditions and parameters used to simulate Cases 1 and 2 (adapted from Helmersen [23]).

Parameter	Case 1	Case 2
Feed flow, $N_{f}$ [mol/s]	$5.513\times 10^{3}$	$3.718\times 10^{-4}$
Feed pressure, $P_{f}$ [$10^{5}$ Pa]	60	5
Permeate pressure, $P_{t}$ [$10^{5}$ Pa]	1.149	1
Temperature bore side, $T_{t}$ [K]	333	298
Temperature shell side, $T_{s}$ [K]	303	298
Fiber length, *L* [m]	0.6	0.8
Fiber outer diameter, $d_{o}$ [µm]	250	180
Fiber inner diameter, $d_{i}$ [µm]	200	126
Number of fibers, $n_{fb}$	$113\times 10^{6}$	2805
Viscosity, η [Pa s]	14.9	14.9

**Table 6:** Feed composition and permeance of the gas mixture of Case 1 (adapted from Helmersen [23]).

Component	Feed Fraction	Permeance
	[mol-%]	[$\frac{\mathrm{mol}}{m^{2}h\;k\;\mathrm{Pa}}$]
${\mathrm{CO}}_{2}$	10.07	0.095
${\mathrm{CH}}_{4}$	77.81	0.002
$C_{2}H_{6}$	7.05	0.001
$C_{3}H_{8}$	3.02	0.001
$C_{4}H_{10}$	1.91	0.001
$C_{5+}$	0.10	0.001
$H_{2}O$	0.03	0.95

**Table 7:** Operational conditions and parameters used to simulate Case 3 (adapted from Pan [9]).

Experimental Conditions	Case 3
Feed pressure, $P_{f}$ [kPa]	5242
Permeate pressure, $P_{t}$ [kPa]	92.8
Molar fraction, *x* [%]	$x_{CH_{4}}=66.05$
	$x_{CO_{2}}=3.38$
	$x_{H_{2}S}=22.09$
	$x_{N_{2}}=8.37$
	$x_{C_{2}H_{6}}=0.11$

**Table 8:** Operational conditions used to simulate Case 4 (adapted from Lock et al. [40]).

Experimental Conditions	Case 4
Feed pressure, $P_{f}$ [kPa]	1500
Permeate pressure, $P_{t}$ [kPa]	101.325
Feed temperature, $T_{s}$ [°C]	37
Feed flow rate, $N_{f}$ [SLPM ^a^]	40
Number of fibers, $n_{fb}$	50
Membrane permeance, $P_{i}[10^{-10}$ mol/(m^2^ · s · Pa)]	$P_{CO_{2}}$ = 73.7
	$P_{CH_{4}}$ = 2.34
	$P_{C_{5}H_{12}}$ = 0.34

^a^ SLPM: Standard Liter per Minute.

**Table 9:** Model validation with experimental data from Lock et al. [40] for permeate stream temperature.

Feed Composition [%]	Experimental	Simulated	U
	[°C]	[°C]	[$W\;\cdot \;m^{-2}\;\cdot \;K^{-1}$]
$x_{CO_{2}}$ = 0; $x_{CH_{4}}$ = 96.2; $x_{C_{5}H_{12}}$ = 3.80	30.67	30.67	0.0123
$x_{CO_{2}}$ = 24.5; $x_{CH_{4}}$ = 72.15; $x_{C_{5}H_{12}}$ = 3.80	30.00	30.00	0.103
$x_{CO_{2}}$ = 48.10; $x_{CH_{4}}$ = 48.10; $x_{C_{5}H_{12}}$ = 3.80	29.25	29.25	0.247
$x_{CO_{2}}$ = 76.96; $x_{CH_{4}}$ = 19.24; $x_{C_{5}H_{12}}$ = 3.80	27.33	27.33	0.413
$x_{CO_{2}}$ = 96.2; $x_{CH_{4}}$ = 0; $x_{C_{5}H_{12}}$ = 3.80	26.00	26.00	0.531

**Table 10:** Operational conditions used to simulate Case 5 (adapted from Coker et al. [26]).

Simulated Conditions	Case 5
Feed pressure, $P_{f}$ [kPa]	5960
Permeate pressure, $P_{t}$ [kPa]	170
Feed temperature, $T_{s}$ [°C]	50
Fiber outside diameter, $d_{o}$ [µm]	300
Fiber inside diameter, $d_{i}$ [µm]	150
Number of fibers, $n_{fb}$	500,000
Active membrane area, *A* [m^2^]	377
Membrane permeance, $P_{i}$ [$10^{-10}$ mol/(m^2^ · s · Pa)]	$P_{CO_{2}}$ = 76.04
	$P_{CH_{4}}$ = 2.34
	$P_{N_{2}}$ = 14.74
	$P_{C_{2}H_{6}}$ = 2.51
	$P_{C_{3}H_{8}}$ = 0.030

## Data Availability

The data supporting the findings of this study are available from the corresponding author upon reasonable request.

## Supplementary Materials

The following supporting information can be downloaded at https://www.mdpi.com/article/10.3390/membranes16040154/s1: Figure S1: Profile of shell side membrane concentrations when considering the Newton’s approach; Figure S2: Profile of shell side membrane concentrations when considering the pseudotransient approach; Figure S3: Profile of fiber-side (tube-side) membrane concentrations when considering the Newton’s approach; Figure S4: Profile of fiber-side (tube-side) membrane concentrations when considering the pseudotransient approach; Figure S5: Mesh convergence analysis for the flow rate of ${\mathrm{CH}}_{4}$ and $C_{2}H_{6}$, Case 1; Figure S6: Mesh convergence analysis for the flow rate of $C_{3}H_{8}$ and $C_{4}H_{10}$, Case 1; Figure S7: Mesh convergence analysis for the flow rate of $C_{5+}$, Case 1.

## Abbreviations

The following abbreviations are used in this manuscript:

BC	Boundary condition
BDF	Backward differentiation formula
CPU	Central processing unit
IC	Initial condition
NMPC	Nonlinear model predictive control
NR	Newton–Raphson
ODE	Ordinary differential equation
PT	Pseudotransient
RK23	Runge–Kutta method of order 2(3)
RK45	Runge–Kutta method of order 5(4)
SS	Steady state
