# Supplementary material for: A Robust Numerical Framework for Hollow-Fiber Membrane Module Simulation and Solver Performance Analysis
Source: Membranes (Basel). 2026 Apr 21;16(4):154. doi: 10.3390/membranes16040154 (PMC13117429; doi:10.3390/membranes16040154)
Supplement: Supplementary file 1 [file membranes-16-00154-s001.zip › membranes-4213541-supplementary.pdf]

Supplementary Material

Manuscript: "A Robust Numerical Framework for Hollow Fiber Membrane Module Simulation and Solver Performance Analysis"

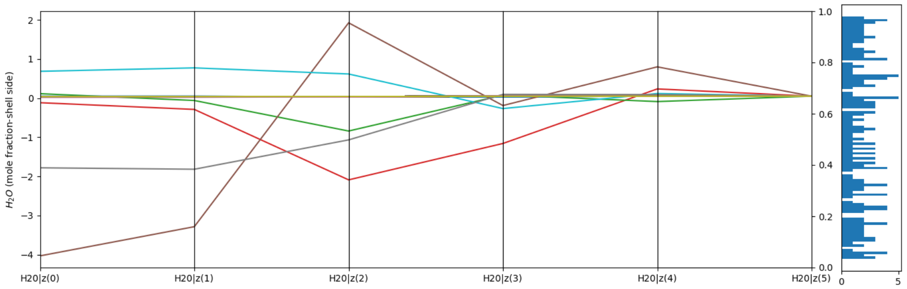

Figure 1: Profile of shell side membrane concentrations when considering the Newton's approach.

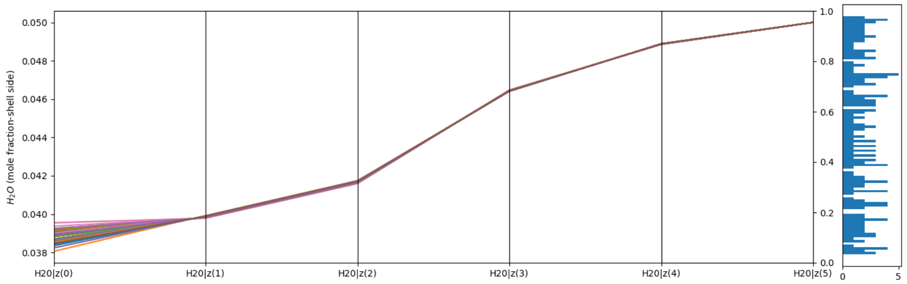

Figure 2: Profile of shell side membrane concentrations when considering the Pseudotransient approach.

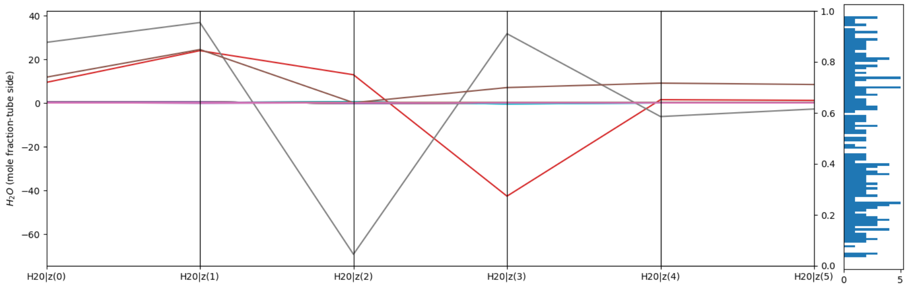

Figure 3: Profile of fiber-side (tube-side) membrane concentrations when considering the Newton's approach.

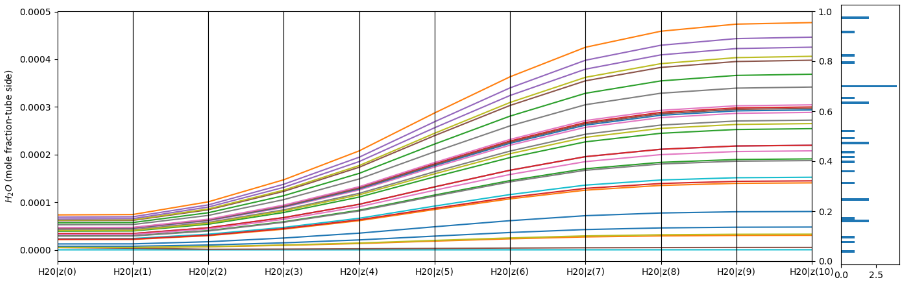

Figure 4: Profile of fiber-side (tube-side) membrane concentrations when considering the pseudo-transient approach.

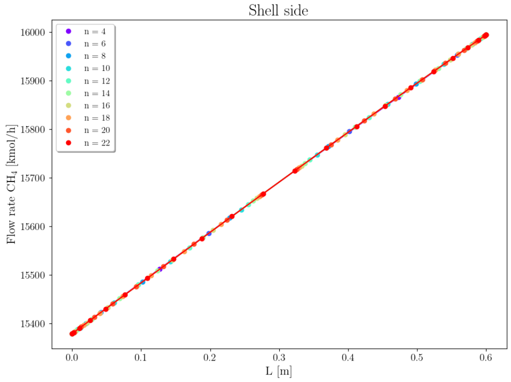

(a) CH_4, shell

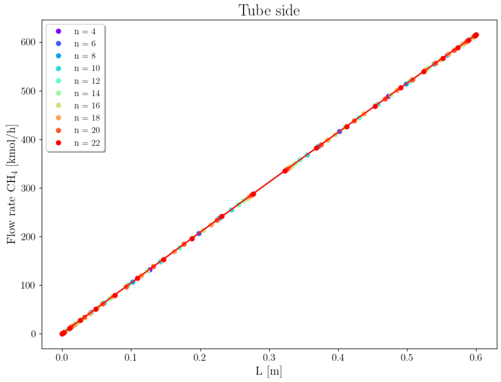

(b) CH_4, fiber

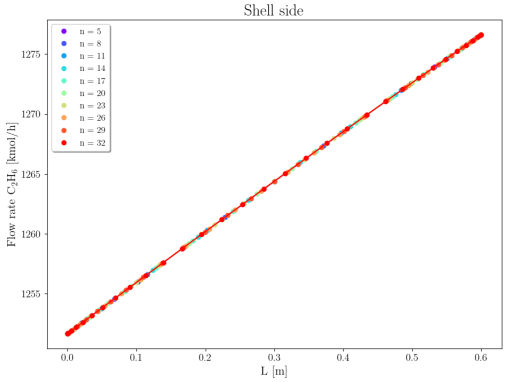

(c) C_2H_6, shell

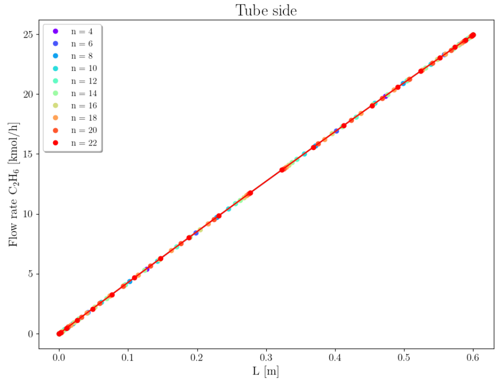

(d) C_2H_6, fiber

Figure 5: Mesh convergence analysis for the flow rate of CH_4 and C_2H_6, Case 1.

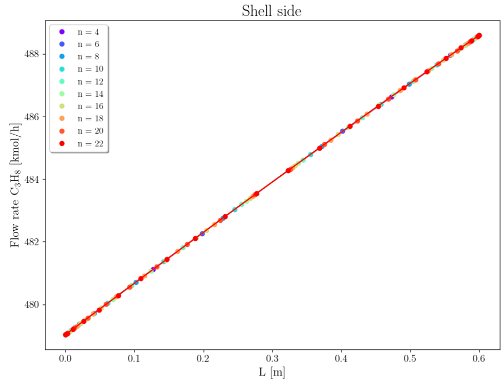

(a) C_3H_8, shell

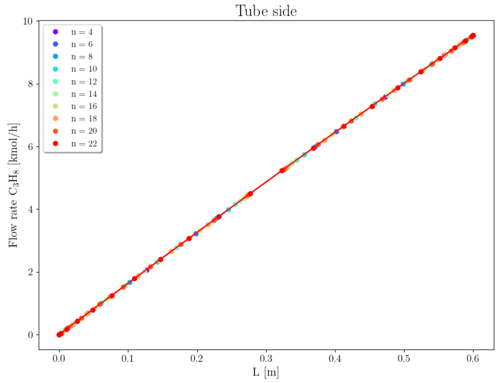

(b) C_3H_8, fiber

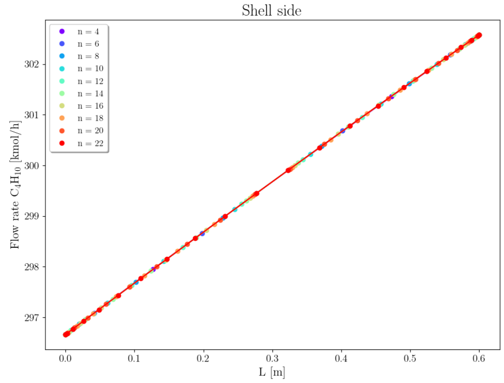

(c) C_4H_{10}, shell

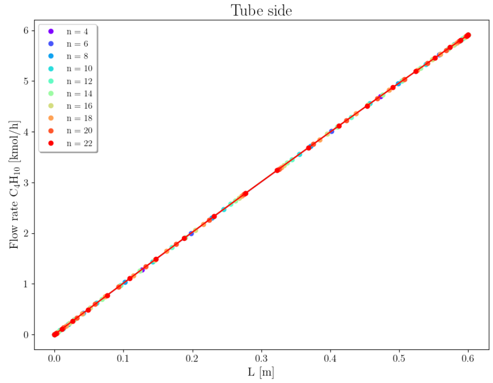

(d) C_4H_{10}, fiber

Figure 6: Mesh convergence analysis for the flow rate of C_3H_8 and C_4H_{10}, Case 1.

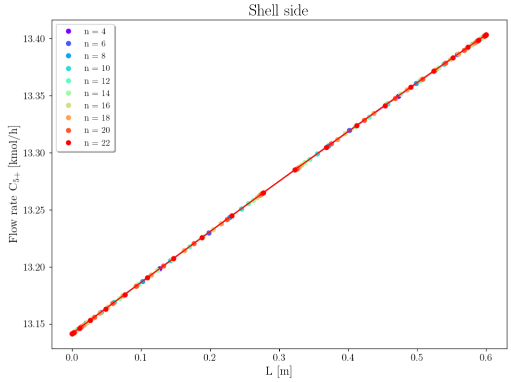

(a) C_{5+}, shell

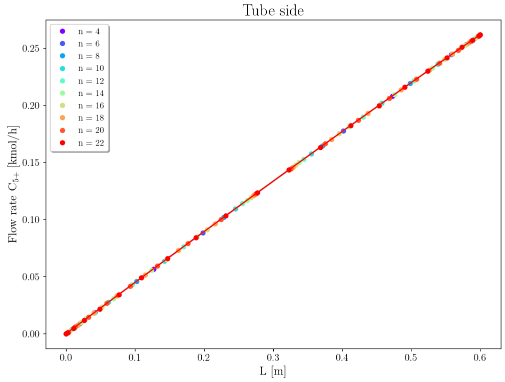

(b) C_{5+}, fiber

Figure 7: Mesh convergence analysis for the flow rate of C_{5+}, Case 1.
